# Improving the precision of shock resuscitation by predicting fluid responsiveness with machine learning and arterial blood pressure waveform data

**DOI:** 10.1038/s41598-023-50120-5

**Published:** 2024-01-26

**Authors:** Chitrabhanu B. Gupta, Debraj Basu, Timothy K. Williams, Lucas P. Neff, Michael A. Johnson, Nathan T. Patel, Aravindh S. Ganapathy, Magan R. Lane, Fatemeh Radaei, Chen-Nee Chuah, Jason Y. Adams

**Affiliations:** 1 Department of Electrical and Computer Engineering, University of California Davis, Davis, CA USA; 2 Wells Fargo, Inc., San Francisco, CA USA; 3 Department of Vascular and Endovascular Surgery, Wake Forest University, Winston-Salem, NC USA; 4 Department of General Surgery, Wake Forest University, Winston-Salem, NC USA; 5 Department of Emergency Medicine, University of Utah, Salt Lake City, UT USA; 6 Meta Platforms, Inc., Menlo Park, CA USA; 7 Division of Pulmonary, Critical Care, and Sleep Medicine, University of California Davis, 4150 V Street, Suite 3400, Sacramento, CA 95817 USA; 8 Department of Computer Science, University of California Davis, Davis, CA USA

**Keywords:** Predictive markers, Predictive medicine, Machine learning

## Abstract

Fluid bolus therapy (FBT) is fundamental to the management of circulatory shock in critical care but balancing the benefits and toxicities of FBT has proven challenging in individual patients. Improved predictors of the hemodynamic response to a fluid bolus, commonly referred to as a fluid challenge, are needed to limit non-beneficial fluid administration and to enable automated clinical decision support and patient-specific precision critical care management. In this study we retrospectively analyzed data from 394 fluid boluses from 58 pigs subjected to either hemorrhagic or distributive shock. All animals had continuous blood pressure and cardiac output monitored throughout the study. Using this data, we developed a machine learning (ML) model to predict the hemodynamic response to a fluid challenge using only arterial blood pressure waveform data as the input. A Random Forest binary classifier referred to as the ML fluid responsiveness algorithm (MLFRA) was trained to detect fluid responsiveness (FR), defined as a ≥ 15% change in cardiac stroke volume after a fluid challenge. We then compared its performance to pulse pressure variation, a commonly used metric of FR. Model performance was assessed using the area under the receiver operating characteristic curve (AUROC), confusion matrix metrics, and calibration curves plotting predicted probabilities against observed outcomes. Across multiple train/test splits and feature selection methods designed to assess performance in the setting of small sample size conditions typical of large animal experiments, the MLFRA achieved an average AUROC, recall (sensitivity), specificity, and precision of 0.82, 0.86, 0.62. and 0.76, respectively. In the same datasets, pulse pressure variation had an AUROC, recall, specificity, and precision of 0.73, 0.91, 0.49, and 0.71, respectively. The MLFRA was generally well-calibrated across its range of predicted probabilities and appeared to perform equally well across physiologic conditions. These results suggest that ML, using only inputs from arterial blood pressure monitoring, may substantially improve the accuracy of predicting FR compared to the use of pulse pressure variation. If generalizable, these methods may enable more effective, automated precision management of critically ill patients with circulatory shock.

## Introduction

Resuscitation of circulatory shock requires judicious management of fluid bolus therapy (FBT) to optimize end-organ perfusion and minimize adverse effects such as end-organ congestion and injury to the endothelial glycocalyx^1^. Multiple studies have documented an association between the volume of fluid administered and adverse outcomes, including death, leading to the concept of “fluid toxicity” from excess administration^2^. Despite this body of research, recent randomized, controlled studies of protocolized, fluid-sparing approaches to shock resuscitation using traditional resuscitation endpoints have failed to show improvements in patient-centered outcomes^3–7^. While several studies have explored novel methods to tailor FBT to specific patient states, critical gaps remain in the armamentarium of methods to optimize the hemodynamic response to FBT for individual patients^8,9^.

Research over the past half century has sought to develop accurate, patient-specific predictors of a favorable hemodynamic response to a fluid challenge, commonly referred to as fluid responsiveness (FR). FR has been defined historically as ≥ 15% increase in cardiac output (CO) in response to an intravenous fluid bolus^10^, although studies of FR have varied by fluid type, administered volume, and the CO threshold used to classify FR^11^. Studies have consistently shown that FR is present only 50% of the time when a fluid bolus was thought to be clinically indicated^12^, highlighting the need for accurate predictors of FR to prevent both under- and over-administration of fluids. Additional predictive methods, particularly those amenable to automated clinical decision support (CDS), are needed to enable delivery of the right dose of FBT, at the right frequency, and at the right phase of a resuscitation to enable personalized hemodynamic optimization^2^.

Predictors of FR can be divided into those requiring active patient intervention, such as the passive leg raise maneuver (PLR), and those calculated from passively-acquired physiologic data such as pulse pressure variation (PPV)^9^. Studies have shown the PLR to discriminate well between FR and non-responsive (NR) states^12^. However, the PLR is time and labor-intensive and requires patient manipulation, specialized beds for proper patient positioning, and measurement of CO before and after the maneuver. These factors limit the ability to incorporate the PLR into automated CDS systems. In contrast, passive metrics like PPV can be reassessed frequently and incorporated into automated CDS. PPV utilizes changes in pulse pressure measured from arterial blood pressure (ABP) waveforms across the respiratory cycle in mechanically ventilated patients to predict FR. Performance of PPV has been variable across studies, ranging from poor to excellent depending on the patient population and clinical setting^8,13^, and its performance may vary over the course of a resuscitation^14^. Suboptimal performance of PPV in critically ill patients has been well-documented in the settings of arrhythmias, low tidal volume ventilation, patient-generated respiratory effort, and poor pulmonary compliance, limiting its widespread use in critical care^15^.

The digital transformation of healthcare presents new opportunities to advance critical care medicine. Increasing integration of medical devices (e.g. bedside physiologic monitors), advanced analytical methods like machine learning (ML), and the democratization of secure cloud computing are facilitating the development of novel predictive algorithms for use in artificial intelligence-enabled CDS^16,17^. To this end, several recent studies have demonstrated the potential of ML models to predict hypotensive events, blood pressure response to FBT, and changes in urine output after fluid resuscitation^18–20^. To expand upon work in this space, we aimed to develop a novel ML-based FR algorithm (MLFRA) and hypothesized that our algorithm would outperform PPV when used to predict FR in large animal models of circulatory shock.

## Methods

### Cohort description

ML model development was performed using ABP data from 394 fluid challenges derived from 58 adult pigs across three injury models of circulatory shock including hemorrhagic shock (n = 134 fluid challenges from 13 pigs), ischemia–reperfusion injury (n = 119 fluid challenges from 13 pigs), and ischemia–reperfusion injury with intermittent variable balloon occlusion of the proximal descending aorta (n = 141 fluid challenges from 32 pigs). All animals were treated with continuous infusion norepinephrine, adjusted to maintain mean arterial pressure (MAP) above 60 mmHg before initiating FBT then locked into a baseline rate. Hemorrhagic shock (HEM) involved controlled hemorrhage of 25% of estimated blood volume. Ischemia–reperfusion injury (IRI) involved controlled hemorrhage followed by 30 min of complete aortic occlusion and then restoration of flow to the lower body^21^. Ischemia–reperfusion injury followed by intermittent occlusion of the supraceliac aorta (EPACC) was performed as previously reported^22^. The HEM and IRI shock models, developed specifically for our MLFRA experiments, were subdivided into hypovolemic, euvolemic, and hypervolemic phases corresponding to pure blood loss, transfusion of the shed blood, and transfusion of an additional 25% blood volume from a donor animal, respectively. During each phase animals received four separate 500 ml boluses of VetivexTM pHyLyteTM solution each delivered over 10 min with a 5-min pause between boluses. Each bolus was administered in micro-boluses of 100 ml over 60 s with 60-s pauses between micro-boluses (Supplementary Fig. 1). EPACC animals were originally treated as part of experiments unrelated to this work but were included in this cohort after our initial experiments with HEM and IRI animals suggested potential benefit from a greater diversity of pathophysiology and resuscitation strategies (see below). EPACC animals were treated with weight-based boluses of VetivexTM pHyLyteTM solution (5 ml/kg) each delivered over 2 min (Supplementary Fig. 1), with titration of both norepinephrine infusion and/or intra-aortic balloon volume determined by a resuscitation algorithm targeting a MAP > 60 and CVP ≥ 5^22^. Boluses from EPACC animals were only included from time periods where both the norepinephrine infusion rate and intra-aortic balloon volume were held constant such that the only factor affecting hemodynamics was the infusion of fluid. All animals were sacrificed using intravenous ethanol (1 ml/10 pounds of body weight) while under general anesthesia. Death was confirmed through electrocardiogram and blood pressure measurements. Additional methods describing the animal experiments can be found in the Methods section of the Supplement. The Institutional Animal Care and Use Committee at Wake Forest Baptist Medical Center approved this study (approval numbers A18-098 and A21-092). All animal experiments were performed and reported in accordance with Animal Research: Reporting of In vivo Experiments (ARRIVE) guidelines and in strict compliance with the Guide for the Care and Use of Laboratory Animals.

### Data acquisition

Waveform data from intra-aortic ABP catheters and CO monitors were collected for 60 s immediately before and after each bolus. CO was measured using either an intra-cardiac pressure–volume (PV) loop catheter or ultrasound flow probe placed over zone 1 of the descending aorta as a surrogate for CO^23^. Stroke volume (SV) was measured by dividing the median CO by the median heart rate during the measurement period. The observed change in SV after each bolus was calculated and used to label boluses as fluid responsive (FR) or fluid non-responsive (NR). FR was defined as a post-bolus increase in SV of ≥ 15%^10,11^.

Physiologic data were visualized using the LabChart™ software platform (ADInstruments, Sydney Australia, version 8.1.19). Waveform data were downsampled from 1000 to 100 Hz. Downsampled data were visually inspected by JYA, DB, and CG and pre-bolus and post-bolus intervals with substantial signal artifacts (e.g., gross motion artifacts, severe signal dampening) were excluded prior to all MLFRA model development to prevent bias from bolus selection (Supplementary Fig. 2). High-frequency noise was removed using a Savitsky-Golay filter (window = 19, polynomial order = 2), followed by labeling of systolic blood pressure, diastolic blood pressure, and the dicrotic notch pressure and their associated timestamps, using “core feature” detection algorithms developed for the study (Supplementary Fig. 3).

### Train/test splitting

To avoid data leakage, we split bolus-associated data at the pig-level for all experiments. Preliminary experiments designed to explore model generalizability involved training on data from a single injury model (e.g., HEM) and testing on pigs from the remaining pathophysiologies. Test performance was inconsistent under these conditions. To assess whether overfitting was the result of small sample sizes in general or to fitting pathophysiology-specific models, we used k-fold cross validation (CV; k = 5) in the training datasets where good performance was observed in the k-folds suggesting models were overfitting to training set pathophysiology and failing to generalize to different pathophysiology rather than overfitting random noise from small sample sizes in general (Supplementary Table 1).

Thus, to test the hypothesis that a more diverse learning space would improve generalizability across different pathophysiologies, subsequent experiments were performed by pooling pigs from all three injury models into a combined dataset. Given our still relatively small dataset (394 boluses from 58 pigs), we used multiple randomly selected train/test splits (n = 29, half of the 58 total pigs) to avoid a biased estimation of model performance from any one random train/test split. We implemented a stratified random pig-level allocation strategy designed to generate train/test splits with a prevalence of FR boluses as close to 50% as possible to reflect the native prevalence reported in the clinical literature^12^. Characteristics of each train/test split can be seen in Supplementary Table 2.

All subsequent model development experiments including algorithm selection, feature selection, hyperparameter tuning, and model training were performed by further splitting the training data using fivefold CV to assess model stability and estimate the anticipated generalization error. Boluses were again split between CV folds at the pig-level using a stratified random allocation attempting to balance FR and NR boluses across folds. Test datasets, also referred to here as “holdout” sets, were not analyzed until all model development experiments were completed. 29 models (one per train/test split) were serialized^24^ and evaluated on the holdout datasets (see section below).

### Feature engineering and selection

ABP waveforms were processed to identify “core features”, which were then used to calculate another set of expert-informed physiologic features similar to prior methods (Supplementary Table 3)^18^. The median and standard deviation of each feature were calculated using all beats from the 60 s prior to each bolus, resulting in a set of 50 features. Four different feature selection mechanisms were compared to select the most informative features, minimize overfitting, and optimize computational requirements. Statistical feature selection used Kolmogorov–Smirnov tests^25^ to retain features with non-overlapping target label distributions (*p* > 0.1), followed by removal of highly correlated features (correlation coefficient threshold ≥ 0.9). Non-statistical methods were assessed including permutation importance^26^, recursive feature elimination^27^ (RFE) and mutual information^28^. The four feature selection methods were applied to each of the 29 training datasets resulting in 116 feature selection trials. RFE showed optimal model performance in cross-validation using 10 features, so all non-statistical selection methods used 10 features to ensure comparability across experiments. One additional method was evaluated where features that were selected in ≥ 50% of the 116 feature selection trials were included to create a consensus feature set (Supplementary Fig. 4).

### Model development and performance assessment

We evaluated four candidate ML algorithms using the Python Scikit-learn software library^24^ (Python version 3.8.3 was used throughout this study) including logistic regression (LR), support vector machine (SVM), random forest (RF), and gradient boosted machine (GBM) algorithms. To minimize overfitting and evaluate the consistency of retained features, we used four different feature selection techniques and k-fold cross-validation method (k = 5) to tune hyperparameters^29^. Model selection targeted the area under the receiver operating curve (AUROC). After all model development experiments were completed in the training sets, models were refitted using all data in each training set and serialized, followed by final testing on the corresponding holdout sets. The primary outcome measure was the AUROC. Models were also evaluated by the area under the precision-recall curve (AUPRC), recall/sensitivity, specificity, precision/positive predictive value, negative predictive value (NPV), and overall accuracy. Confusion matrix-based measures used a model decision threshold of ≥ 50% but models were also evaluated across deciles of decision threshold to better understand the range of performance. As a comparative benchmark, the performance of pulse pressure variation (PPV) was evaluated in the holdout datasets using the same metrics. PPV was calculated from ABP data in the 60-s period immediately prior to each fluid bolus by dividing the difference between the largest and smallest pulse pressures by the mean of the largest and smallest pulse pressures^15^. PPV was evaluated using AUROC and using a threshold of ≥ 12% for confusion matrix-based measures^13^. Metrics were reported with 95% confidence intervals calculated using all 29 holdout sets; results of individual train/test splits are also reported in the Supplementary Table 4. We followed the transparent reporting of a multivariable prediction model for individual prognosis or diagnosis (TRIPOD) recommendations for evaluating the prediction models (Supplementary Table 5)^30^.

### Error analysis

To investigate systematic contributors to misclassification in the holdout datasets, we examined several factors. First, we looked to see if misclassifications were more common when the change in SV after FBT was near the defined FR boundary condition. In this regard, use of a target label defined by a statistically-derived threshold value of a continuous variable^10^ can challenge the development of a binary classifier^31^. We thus classified boluses into three subgroups, one in the grey zone encompassing the 15% threshold used to define FR (change in SV of 10–20%), a < 10% change group, and a > 20% group and examined the proportion of grey zone boluses as a function of model performance (by AUROC). Next, we looked for an association between performance and injury model by plotting the proportion of boluses from each injury model in each holdout dataset against each MLFRA model’s AUROC. Finally, because of our relatively small dataset size, we looked to see if model performance might be related to having randomly split the data into particularly similar or dissimilar train/test splits. We thus used two-sample Kolmogorov–Smirnov tests^25^ (using an a priori *p* value of > 0.1) to identify features with non-overlapping distributions in each train/test split and plotted the proportion of non-overlapping features per split against the AUROC. Best fit lines (using Python’s NumPy library^32^) and Pearson correlation coefficients (using Python’s SciPy library^33^) were used to describe the relationships described above.

## Results

The average number of boluses allocated to training and test splits across all 29 pig-level dataset splits, including the proportion of FR boluses and the proportion of boluses derived from each injury model is described in Table 1. Our stratified random sampling approach was able to achieve a near 50% proportion of FR boluses^12^ and roughly equal proportions of boluses derived from each injury model despite different numbers of pigs from each injury model, different numbers of boluses from each pig, and different proportions of FR boluses from each pig in the overall dataset. Details of each train/test split can be seen in Supplementary Table 2 and hemodynamic variables including the median change in stroke volume after a fluid challenge can be seen in Supplementary Table 6.

**Table 1:** Number of boluses in training and test datasets overall, and proportions by fluid responsiveness and source injury model across all 29 pig-level train/test dataset splits.

Training set (n)	Test set (n)	% FR, training	% FR, test	% IRI, training	% EPACC, training	% HEM, training	% IRI, test	% EPACC, test	% HEM, test
269	125	58	58	30	34	36	30	40	30

*FR* fluid responsive, *IRI* ischemia–reperfusion, *EPACC* endovascular perfusion augmentation for critical care, *HEM* hemorrhage.

Results of CV experiments in the training data showed comparable performance of the RF, GBM, LR, and SVM models (Supplementary Table 7). Due to its simplicity and inherent tendency to resist overfitting^34^, all subsequent experiments were performed using the RF algorithm. Comparative feature selection experiments using CV in the training data showed similar performance between methods; results in the holdout test sets are reported in Table 2 and also showed comparable performance across methods. Supplementary Fig. 5 shows the list of features retained in ≥ 50% of models across the 4 feature selection methods along with their SHAP values^35^.

**Table 2:** Classification performance metrics for machine learning-based prediction of fluid responsiveness in the 29 holdout datasets across different feature selection methods.

Feature selection method	Number of retained features	Accuracy	AUROC	Precision	Recall	Specificity	AUPRC
Statistical feature selection	18*	0.77 ± 0.01	0.84 ± 0.02	0.77 ± 0.02	0.86 ± 0.02	0.64 ± 0.04	0.85 ± 0.02
RFE	10	0.76 ± 0.01	0.82 ± 0.01	0.76 ± 0.01	0.86 ± 0.03	0.62 ± 0.04	0.84 ± 0.01
Permutation Importance	10	0.75 ± 0.02	0.82 ± 0.02	0.75 ± 0.01	0.86 ± 0.03	0.60 ± 0.04	0.85 ± 0.01
Mutual Information	10	0.76 ± 0.01	0.82 ± 0.02	0.76 ± 0.02	0.86 ± 0.03	0.62 ± 0.04	0.84 ± 0.02
Top 12 Features (> 50% Frequency)	12	0.76 ± 0.02	0.82 ± 0.02	0.77 ± 0.02	0.86 ± 0.02	0.64 ± 0.03	0.83 ± 0.02

*AUROC* Area under receiver operating characteristic curve, *AUPRC* area under precision recall curve.

*For statistical feature selection, this value is the mean of the number of features retained across the 29 holdout datasets.

**Figure 1:**
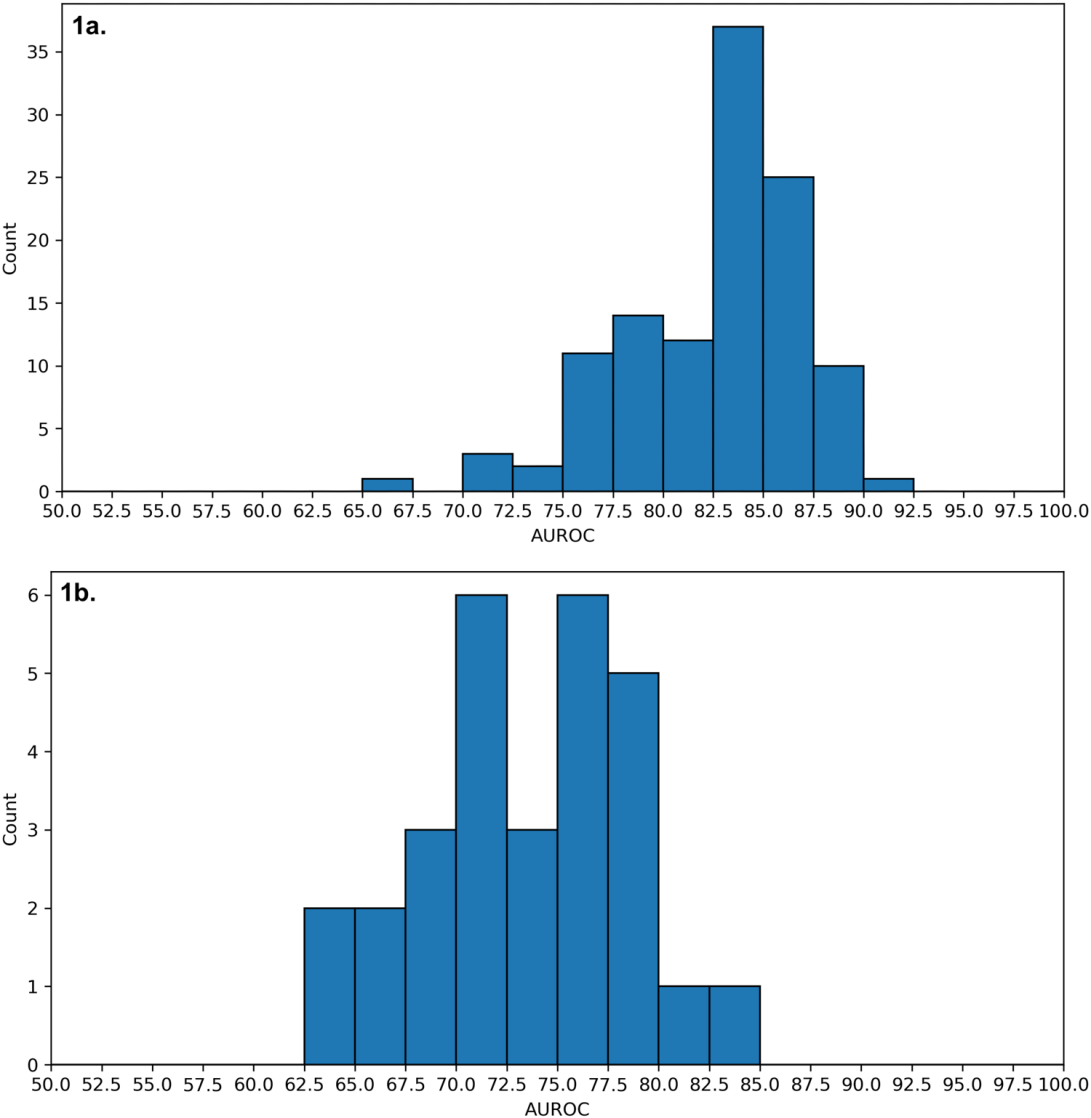
Distribution of area under receiver operating characteristic curve (AUROC) values for machine learning-based fluid responsiveness prediction in the holdout datasets for all 29 pig splits using all four feature selection methods (**a**) and in the same 29 holdout datasets for pulse pressure variation (PPV) based prediction (**b**).

Results in both the training and holdout test datasets showed higher AUROC for the MLFRA than for PPV in discriminating between FR and non-FR boluses (Supplementary Table 8). Figure 1 and Supplementary Tables 4 and 8 show consistently higher performance of the MLFRA compared to PPV albeit with a wider distribution of AUROCs across MLFRA models and experimental conditions. We also evaluated our MLFRA models with multiple confusion matrix statistics at a prediction threshold of ≥ 0.5 and compared this to the commonly used PPV threshold of ≥ 12%. Results in Supplementary Table 8 showed lower sensitivity (i.e., recall) of the MLFRA models compared to PPV but higher average specificity, precision (i.e., positive predictive value), and overall accuracy. Table 3 shows the performance of the MLFRA as assessed by confusion matrix statistics across deciles of model classification threshold.

**Table 3:** Model performance characteristics across a range of predicted probability thresholds using results from the 12-feature prediction model averaged across all 29 holdout datasets.

Threshold	Accuracy	Precision	Sensitivity	Specificity
0	0.58	0.58	1	0
0.1	0.60	0.60	0.99	0.08
0.2	0.70	0.67	0.96	0.35
0.3	0.74	0.71	0.94	0.46
0.4	0.75	0.73	0.91	0.55
0.5	0.77	0.77	0.86	0.64
0.6	0.78	0.80	0.83	0.71
0.7	0.76	0.82	0.77	0.76
0.8	0.70	0.83	0.61	0.83
0.9	0.48	0.83	0.13	0.96
1	0.42	NA*	0	1

*Precision is NA since there were no positive classifications at that threshold.

**Figure 2:**
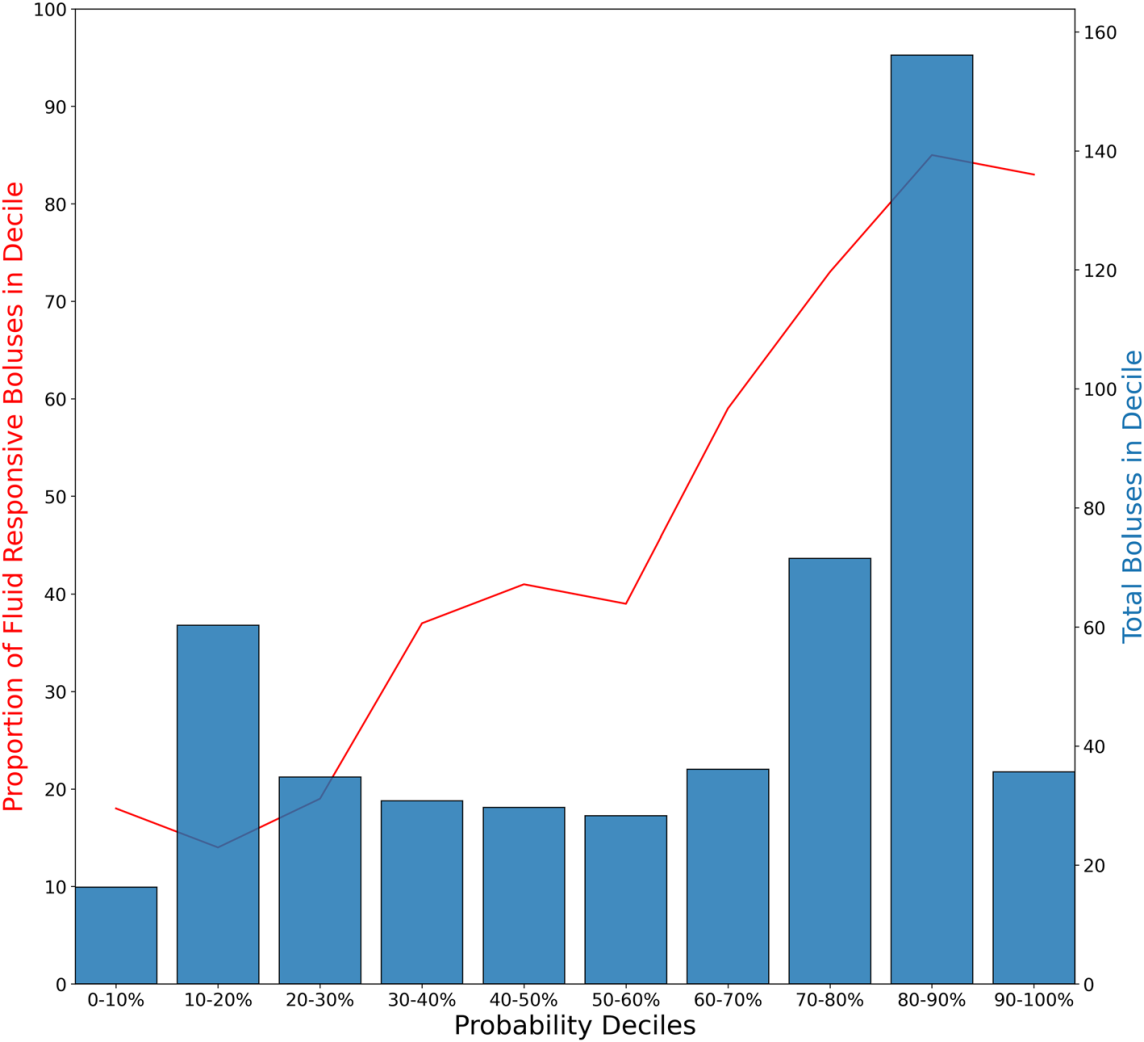
Model calibration curve. Calibration curve for machine learning models trained on the 29 pig splits across all 4 feature selection methods and evaluated on the corresponding holdout datasets.

In addition to characterizing MLFRA model discrimination, we also examined model calibration. Figure 2 shows the predicted probability of FR for each bolus in the holdout datasets, grouped into deciles of predicted probability, against the proportion of fluid responsive boluses in each corresponding decile and the number of boluses in each decile. While the number of boluses in each decile was not uniform, the MLFRA appeared to be well-calibrated across the range of model predictions.

Our error analysis showed a moderately negative correlation (correlation coefficient of − 0.6) between the AUROC of each model and the proportion of boluses with a change in SV between 10 and 20% in each corresponding holdout dataset (Supplementary Fig. 6). Analysis showed weak correlations between model performance and the proportion of pigs from each injury model allocated to each holdout dataset, with correlation coefficients for the proportion of HEM, IRI, and EPACC pigs of -0.15, 0.19, and -0.03 (Supplementary Fig. 7–9). Finally, despite our use of multiple random splits of the overall dataset to mitigate the effects of selection bias on performance estimates, we evaluated whether model performance was related to the degree of dissimilarity in input feature distributions between training and test splits generated by our stratified random splitting method. We observed a weak correlation (− 0.15) between model performance and the proportion of input features with statistically different distributions between the training and holdout test sets of each of the 29 dataset splits (Supplementary Fig. 10).

## Discussion

In this study, we developed a novel approach to the prediction of FR using classical ML methods and only ABP waveform data as input. Our ML fluid responsiveness algorithm (MLFRA) demonstrated good discrimination between FR and NR states (average AUROC 0.82 across different modeling approaches) and performed substantially better than PPV (average AUROC 0.73), a widely used automated FR prediction method and resulted in good model calibration across deciles of predicted probabilities. While our total dataset size was relatively small (n = 394 boluses from 58 pigs), MLFRA models performed consistently well across dataset splits and different ML modeling approaches.

As healthcare undergoes a rapid digital transformation, algorithm driven CDS systems will be used to optimize patient outcomes, reduce costs, and improve patient and provider experience in multiple domains including critical care and resuscitation medicine^16,17,36^. In this context, our MLFRA performed well compared to the two most well-studied predictors of FR – PLR and PPV—with several potential advantages. Across meta-analyses, PLR has performed consistently well in predicting FR with AUROC ranging from 0.84–0.96 when using a change in CO or SV as the FR metric^8,12,37,38^. Despite excellent predictive characteristics, performing the PLR properly is labor intensive, time consuming, requires specialized beds and CO monitoring, and may be contraindicated in highly unstable patients thus prohibiting its use in automated CDS systems or where resources are unavailable^39^.

Like our MLFRA, PPV uses ABP waveform data as input and requires no patient intervention or other monitoring making it suitable for automated CDS. Unlike the PLR, PPV’s reported performance has varied considerably across studies, ranging from poor to excellent, with suboptimal performance in circumstances common in critically ill patients including arrhythmias, poor respiratory system compliance, and when the tidal volume (TV) is < 8 ml/kg of predicted body weight (PBW)^8,13,15^. While ventilator management in our study was ultimately at the discretion of the treating team, lung protective ventilation was recommended including TV of 6–8 ml/kg of PBW and a low PEEP-FiO2 strategy^40^. In this context, our finding that the MLFRA performed consistently better than PPV across dataset splits and feature selection methods suggests that our approach may be more performant than PPV across a broader range of clinical conditions encountered in the intensive care unit (Supplementary Tables 4 and 8)^41^. It is also notable that all features retained in > 50% of 116 dataset splits and feature selection approaches were standard deviation-based features suggesting that MLFRA models were learning to predict FR using indicators of cardiopulmonary interactions over the respiratory cycle similar to PPV^15^. It remains unclear whether the MLFRA’s performance advantages over PPV resulted from use of multiple hemodynamic indicators of cardiopulmonary interactions (versus PPV’s univariate approach) or from the combined use of features representing cardiopulmonary variability and absolute values. Additional studies will need to be performed to determine if the MLFRA consistently outperforms PPV across a broader range of conditions known to compromise PPV performance^14,15^.

Our findings extend recent work applying ML to predicting hemodynamic trajectories and the response to FBT. Bataille et. Al.^42^ used ML to predict FR using features derived from echocardiography in 100 patients with sepsis. While performance was comparable to PLR, the acquisition of echocardiographic data required active intervention from experts, hindering use in automated CDS. Several other recent studies have applied ML methods using data from the Medical Information Mart for Intensive Care database to predict the blood pressure^20^ and urine output^19^ response to FBT. ML operating on ABP waveform data has also been used to predict hypotensive episodes in both ICU and operative patients up to 15 min prior to an event^18^. These studies highlight the potential of learning algorithms to predict hemodynamic trajectories and the response to FBT. To our knowledge, no other study to date has shown the ability of ML to predict the cardiac response to FBT using passively collected ABP waveform data.

Our study has several limitations. First, while our sample size is large for large animal resuscitation studies, it is relatively small compared to many clinical studies. In this regard, we attempted to maximize use of available data by training and testing our MLFRA with three different critical illness models and multiple feature selection methods, and characterized performance using multiple dataset splits to minimize sampling bias and multiple measures of both model discrimination and calibration. While our error analysis did not find clear reasons for FR misclassification other than the proportion of boluses near the SV boundary condition (Supplementary Fig. 6–9), additional large animal studies involving a broader range of clinical conditions will be necessary to better understand the MLFRA’s strengths and weaknesses across disease states and approaches to resuscitation (e.g., type of shock, depth of shock, fluid conservative versus fluid liberal strategies). Ultimately, carefully conducted clinical studies will be necessary to understand how well the MLFRA translates to the bedside under real-world conditions. Second, we recorded ABP tracings measured directly from the femoral artery (HEM and IRI) or aorta (EPACC) and it is possible that our results could be different at other sites of measurement, where differences in ABP waveform morphology could potentially affect input feature calculations. Additional experiments to determine whether our MLFRA continues to outperform PPV across different sites of ABP measurement will be important to determine generalizability to clinical practice. Similarly, our selection of ABP-based input features was not exhaustive, and it is possible that performance would improve with use of a different feature set or the use of deep learning algorithms that don’t require expert feature design. Third, we only compared the MLFRA to PPV and not to other predictors such as the PLR^15^ given the inability to incorporate these predictors into automated CDS systems, and it’s possible that some could have outperformed our MLFRA. Fourth, we developed the MLFRA as a binary classifier. Like PPV, this classification scheme may not perform well around the SV threshold used to separate FR and NR states^10,41^. Our error analysis findings showing an inverse relationship between MLFRA model performance and the proportion of boluses near the SV threshold used to define FR (Supplementary Fig. 6) support this hypothesis, and it is possible that a multi-class classifier trained on minimally-responsive, marginally-responsive, and highly-responsive states, or a regression model predicting the expected change in SV might perform better and provide more clinically-relevant information. In this regard, we selected a 50% voting threshold for the classification of FR by the Random Forest model to enable consistent model evaluation across experimental conditions, and it is possible that a lower or higher threshold of classification (Table 3) might be more desirable at different time points in a resuscitation rather than a “one size fits all” approach to predicting FR^2,14^. Finally, the limitations of the historical definition of fluid responsiveness must be considered. We used a 15% increase in SV to define fluid responsiveness. This commonly used boundary condition is based on the limits of precision of measuring the cardiac response to FBT^10^ and may not necessarily correlate with improvements in tissue perfusion. Future research should explore the ability to predict FR defined by improved end-organ perfusion rather than changes in SV or CO alone.

In conclusion, we report the development of a novel ML model to predict the SV response to FBT using ABP waveform data as the sole input. Our model outperformed pulse pressure variation – a widely used predictor of FR – in multiple injury models of circulatory shock. Additional research is needed to understand the generalizability of our approach in a broader range of disease states and to develop models that predict FBT-mediated improvements in end-organ function rather than hemodynamics alone. Incorporation of such models into automated clinical decision support systems will ultimately enable providers to maximize the benefits of FBT, minimize risks of fluid toxicity, and enable precision resuscitation.

### Supplementary Information

Supplementary Figure S1.

Supplementary Figure S2.

Supplementary Figure S3.

Supplementary Figure S4.

Supplementary Figure S5.

Supplementary Figure S6.

Supplementary Figure S7.

Supplementary Figure S8.

Supplementary Figure S9.

Supplementary Figure S10.

Supplementary Table S11.

Supplementary Table S12.

Supplementary Table S13.

Supplementary Table S14.

Supplementary Table S15.

Supplementary Table S16.

Supplementary Table S17.

Supplementary Table S18.

Supplementary Table S19.

## Data Availability

The datasets used and/or analyzed during the current study are available from the corresponding author on reasonable request.
