# Supplementary figures and images for: Improving the precision of shock resuscitation by predicting fluid responsiveness with machine learning and arterial blood pressure waveform data
Source: Sci Rep. 2024 Jan 26;14:2227. doi: 10.1038/s41598-023-50120-5 (PMC10817926; doi:10.1038/s41598-023-50120-5)

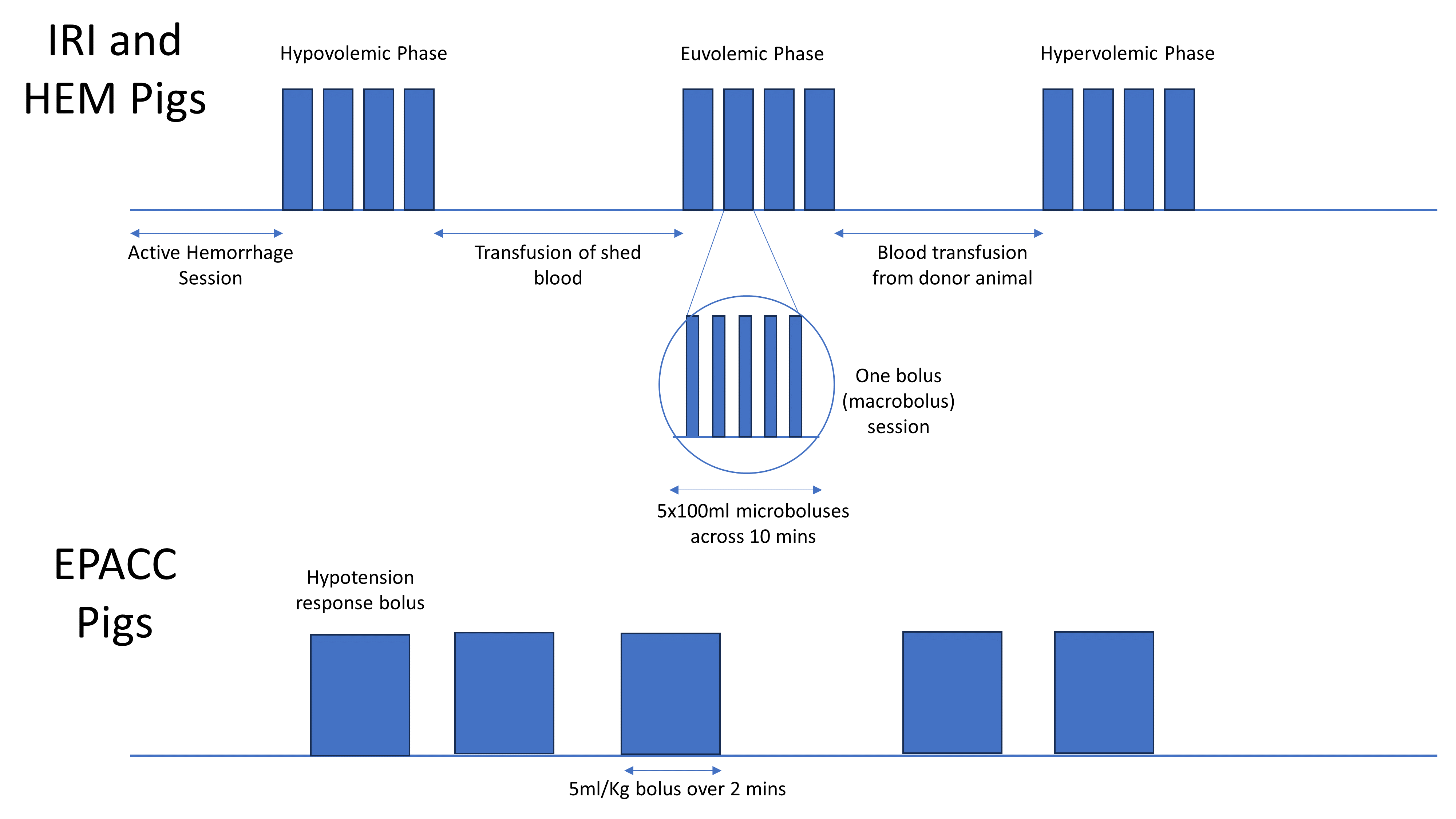

Supplement: Supplementary file 1 — Supplementary Figure S1. [file 41598_2023_50120_MOESM1_ESM.png]

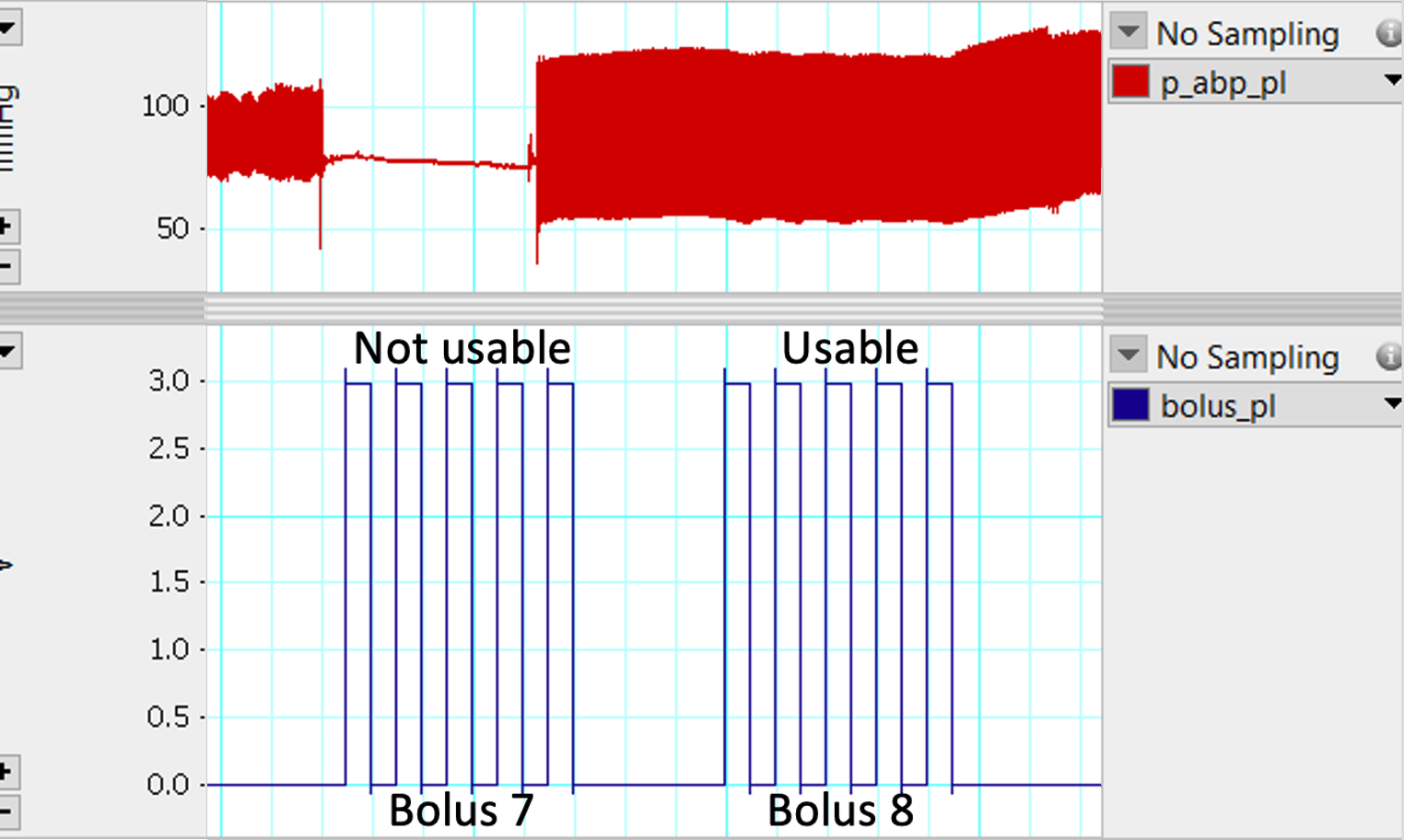

Supplement: Supplementary file 2 — Supplementary Figure S2. [file 41598_2023_50120_MOESM2_ESM.png]

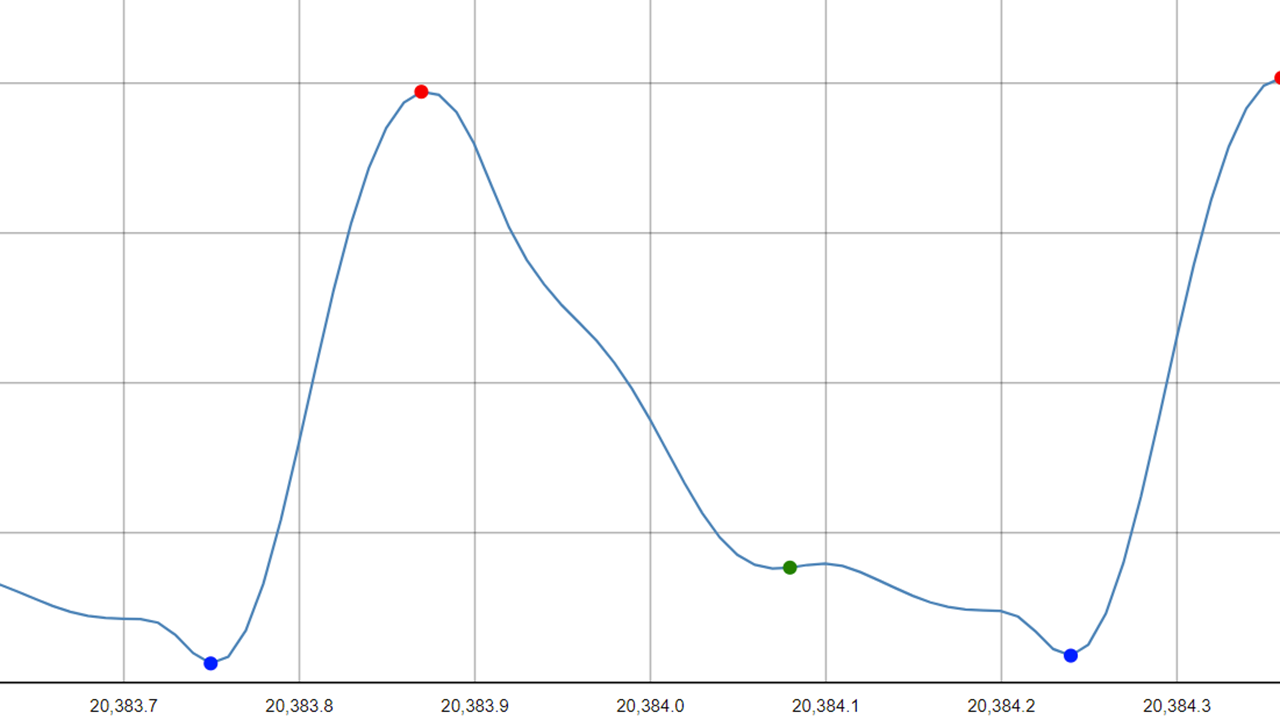

Supplement: Supplementary file 3 — Supplementary Figure S3. [file 41598_2023_50120_MOESM3_ESM.png]

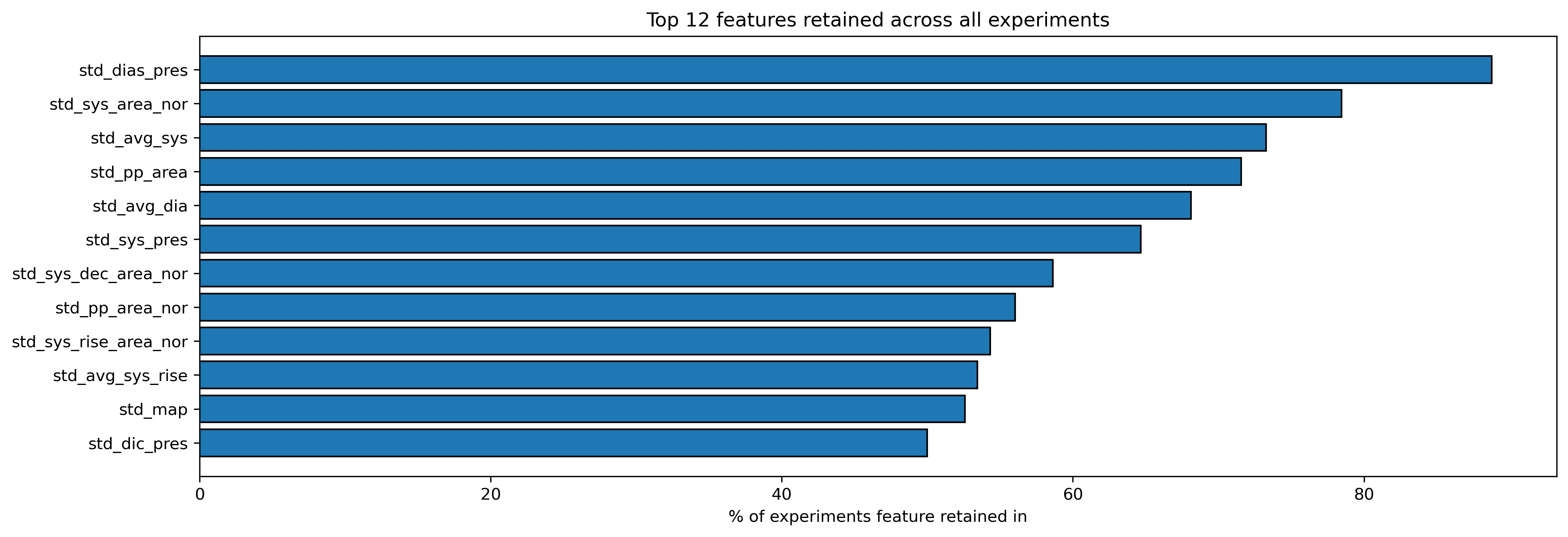

Supplement: Supplementary file 4 — Supplementary Figure S4. [file 41598_2023_50120_MOESM4_ESM.png]

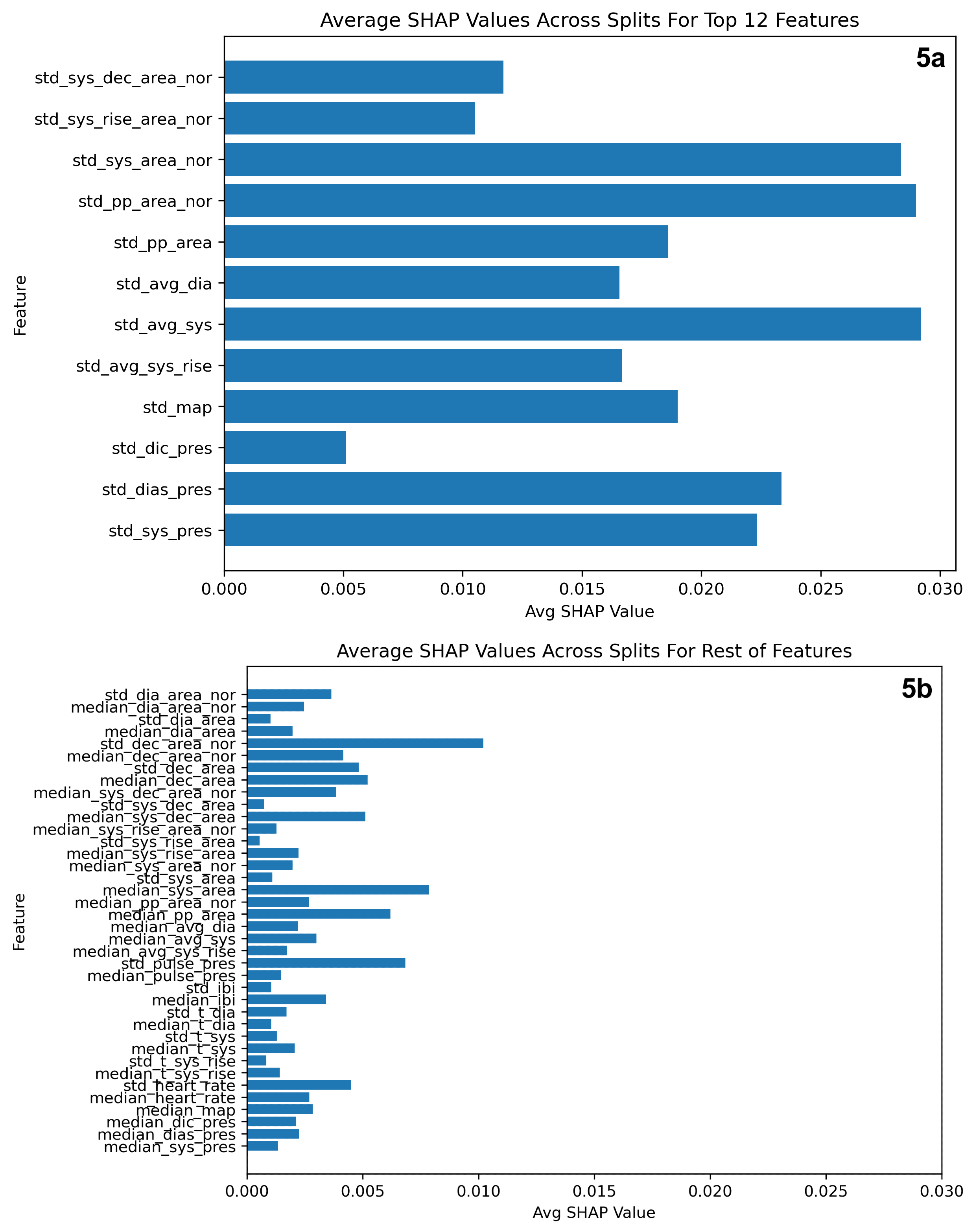

Supplement: Supplementary file 5 — Supplementary Figure S5. [file 41598_2023_50120_MOESM5_ESM.png]

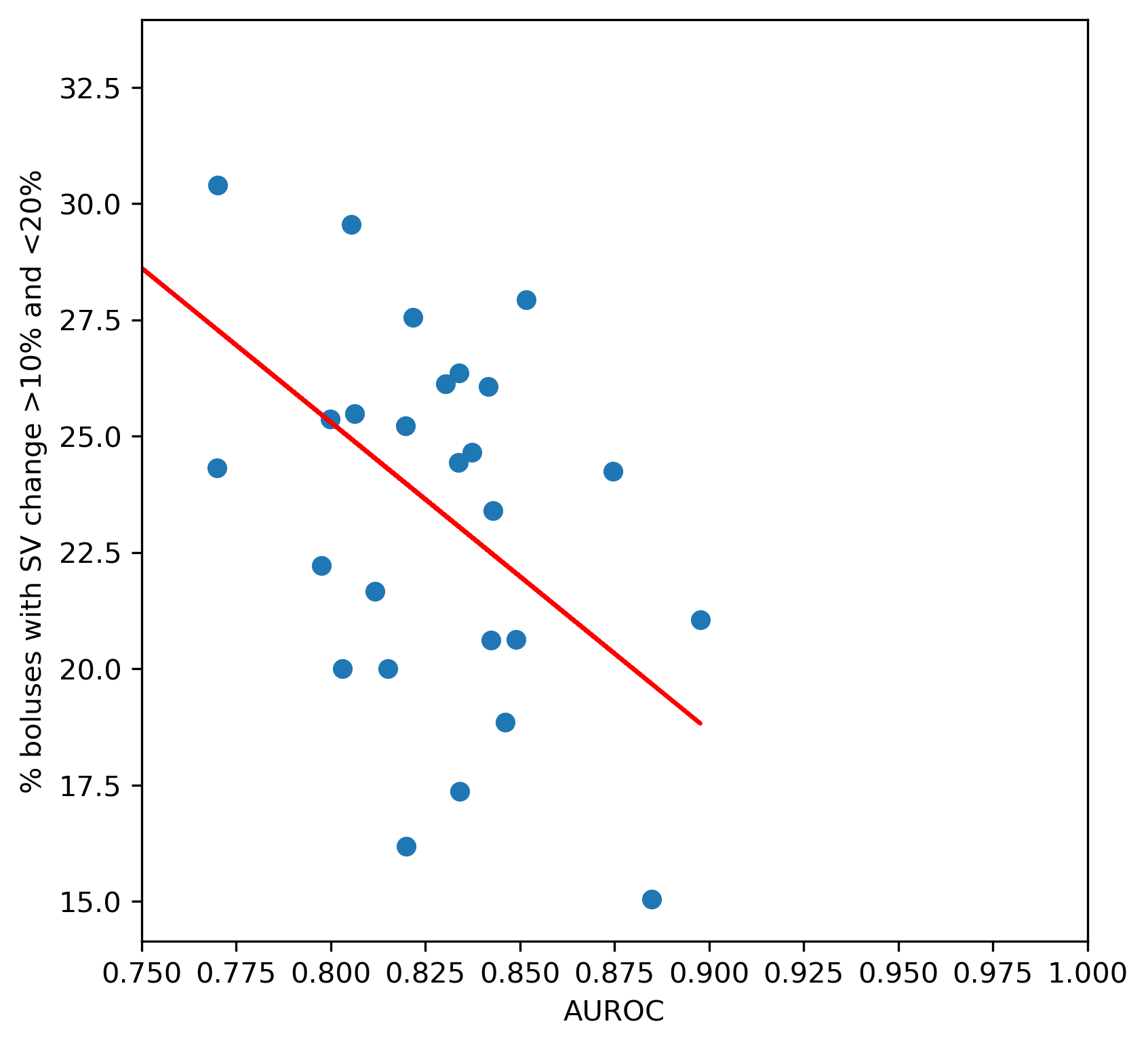

Supplement: Supplementary file 6 — Supplementary Figure S6. [file 41598_2023_50120_MOESM6_ESM.png]

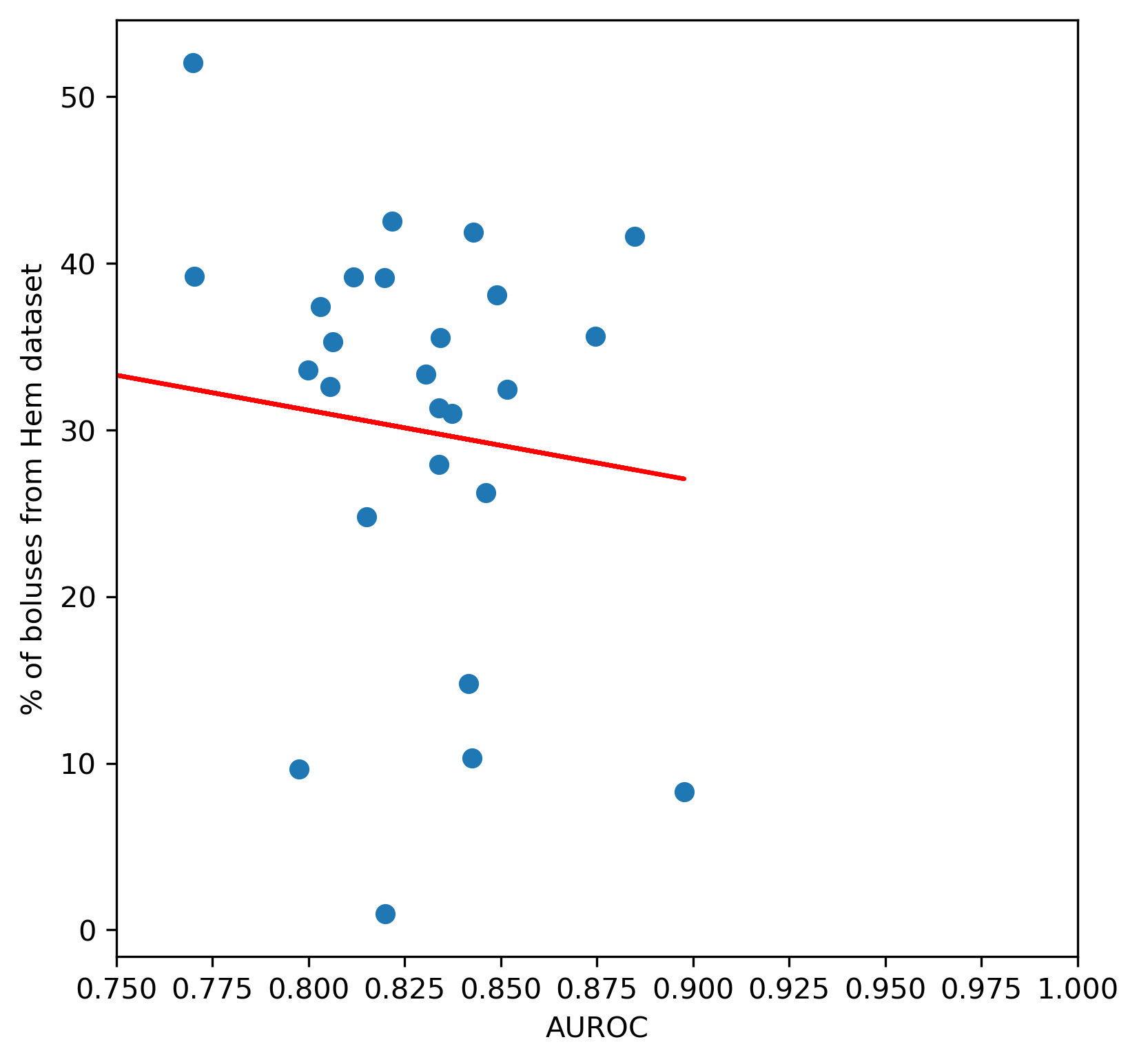

Supplement: Supplementary file 7 — Supplementary Figure S7. [file 41598_2023_50120_MOESM7_ESM.png]

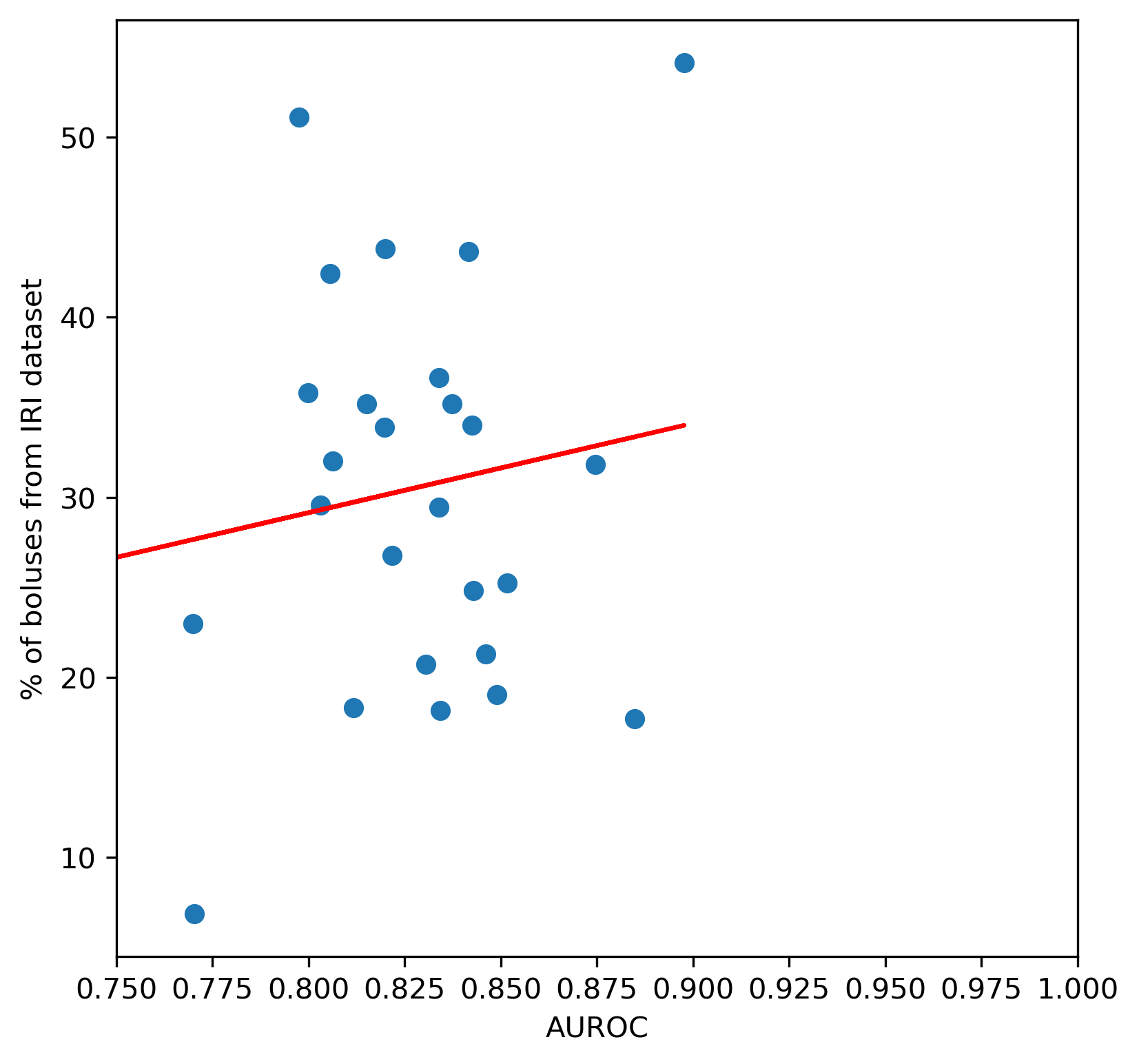

Supplement: Supplementary file 8 — Supplementary Figure S8. [file 41598_2023_50120_MOESM8_ESM.png]

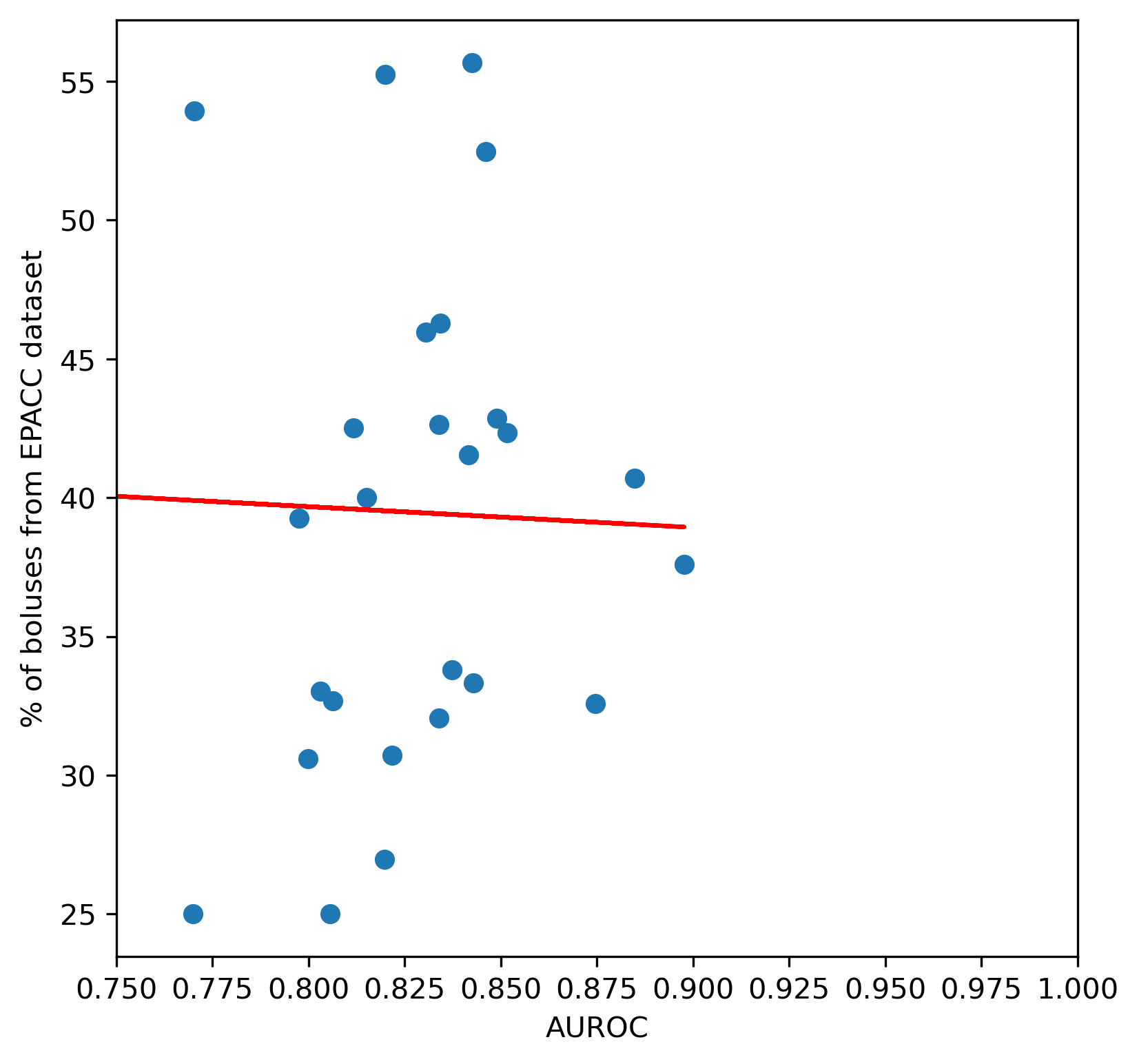

Supplement: Supplementary file 9 — Supplementary Figure S9. [file 41598_2023_50120_MOESM9_ESM.png]

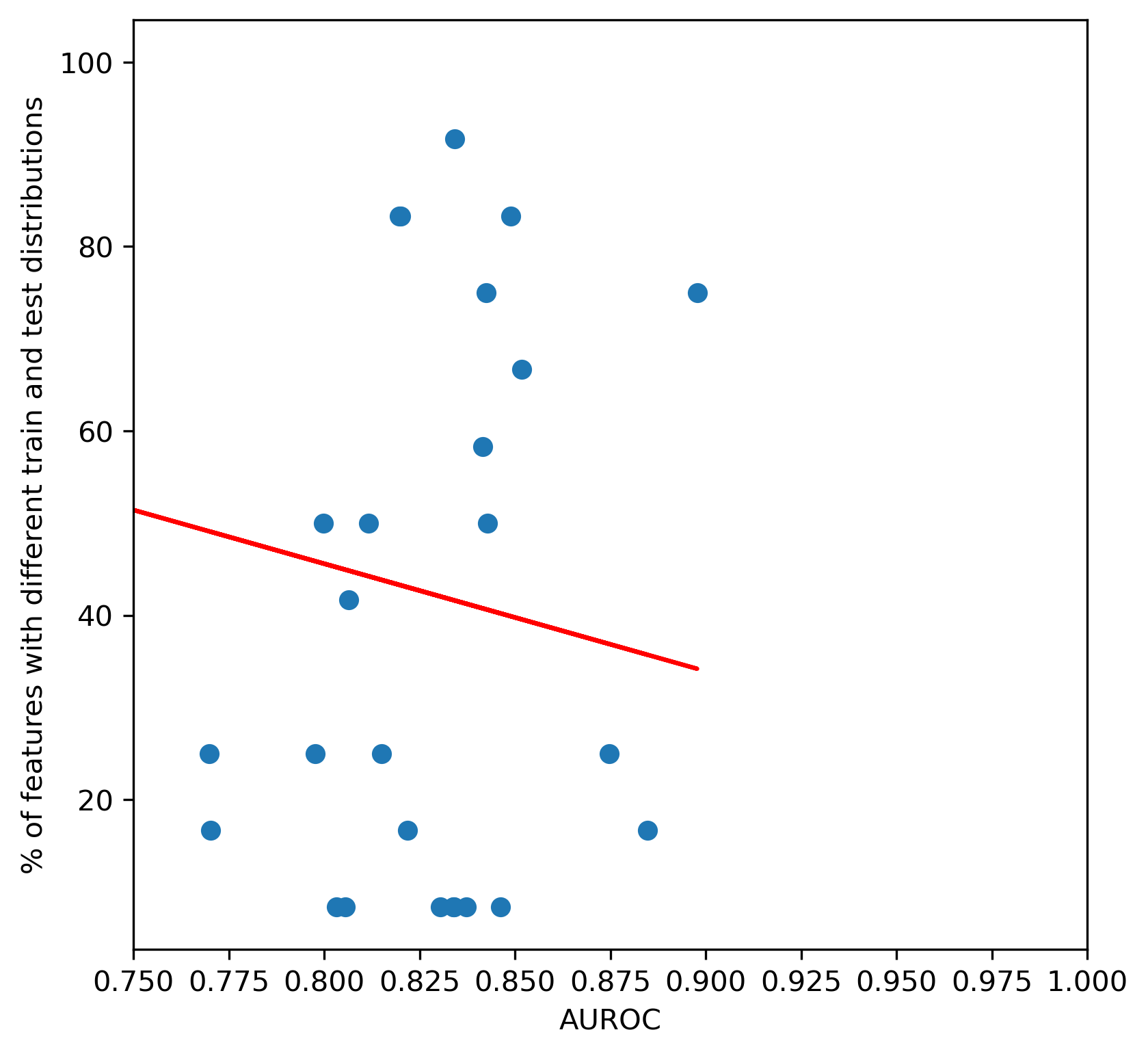

Supplement: Supplementary file 10 — Supplementary Figure S10. [file 41598_2023_50120_MOESM10_ESM.png]

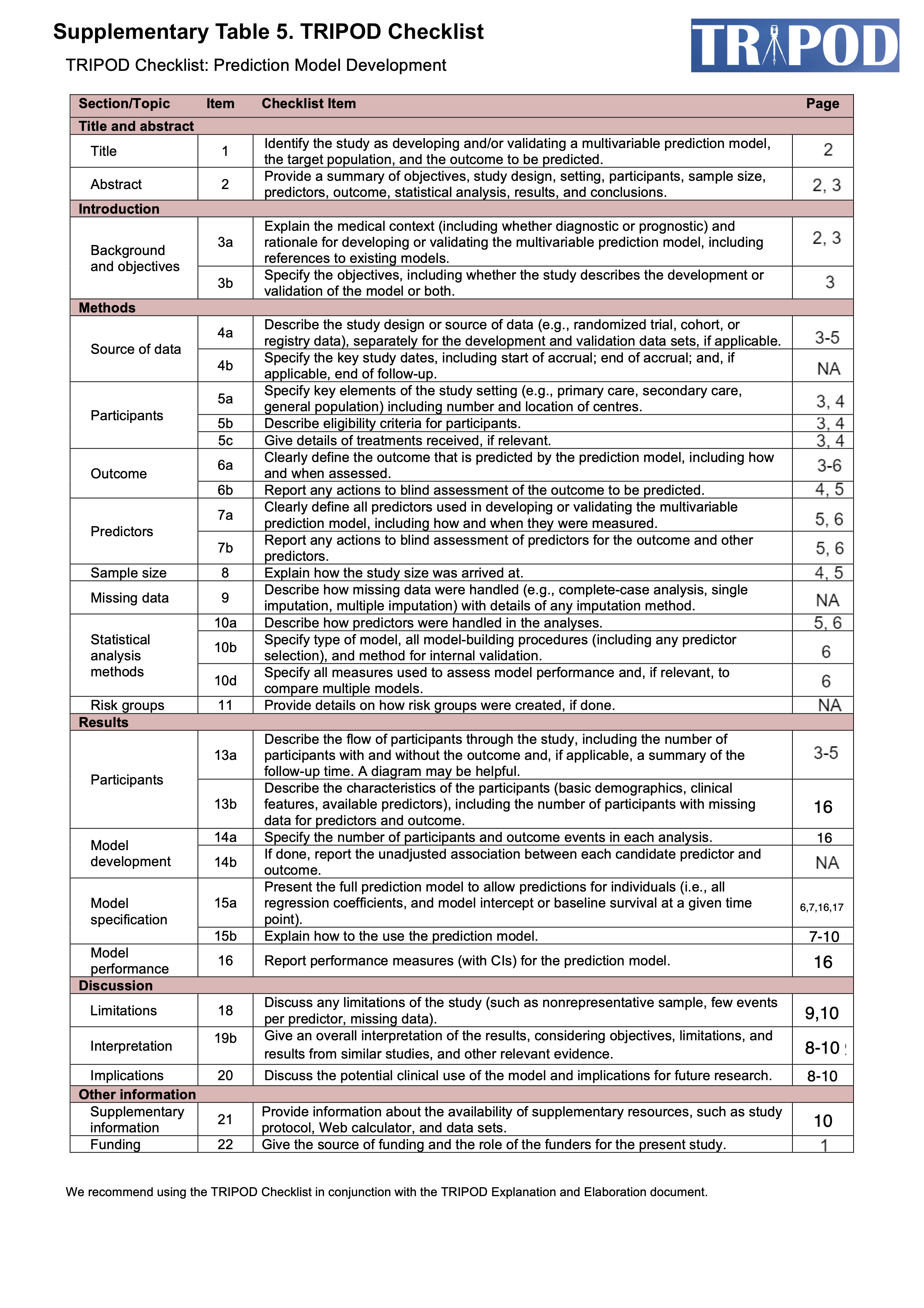

Supplement: Supplementary file 15 — Supplementary Table S15. [file 41598_2023_50120_MOESM15_ESM.png]
