# Supplementary material for: Improving the precision of shock resuscitation by predicting fluid responsiveness with machine learning and arterial blood pressure waveform data
Source: Sci Rep. 2024 Jan 26;14:2227. doi: 10.1038/s41598-023-50120-5 (PMC10817926; doi:10.1038/s41598-023-50120-5)
Supplement: Supplementary file 19 — Supplementary Table S19. [file 41598_2023_50120_MOESM19_ESM.docx]

# **Supplementary Content**

# **Improving the Precision of Shock Resuscitation by Predicting Fluid Responsiveness with Machine Learning and Arterial Blood Pressure Waveform Data**

**Chitrabhanu Gupta^1*^, Debraj Basu, PhD^2*^, Timothy K. Williams, MD^3^, Lucas P. Neff, MD^4^, Michael A. Johnson, MD PhD^5^, Nathan T. Patel, MD^4^, Aravindh S. Ganapathy, MD^4^, Magan R. Lane, BS^4^, Fatemeh Radaei, MS^6^, Chen-Nee Chuah, PhD^1^, Jason Y. Adams, MD MS^7^**

**^1^Department of Electrical and Computer Engineering, University of California Davis, Davis, CA**

**^2^Wells Fargo, Inc., San Francisco, CA****

**^3^Department of Vascular and Endovascular Surgery, Wake Forest University, Winston-Salem, NC**

**^4^Department of General Surgery, Wake Forest University, Winston-Salem, NC**

**^5^Department of Emergency Medicine, University of Utah, Salt Lake City, UT**

**^6^Meta Platforms, Inc., Menlo Park, CA*****

**^7^Division of Pulmonary, Critical Care, and Sleep Medicine, University of California Davis, Sacramento, CA**

***These authors contributed equally to this work**

****Study research performed while a post-doctoral scholar in the Department of Department of Electrical and Computer Engineering, University of California Davis, Davis, CA**

*****Study research performed while a Masters degree student in the Department of Computer Science, University of California Davis, Davis, CA**

**Corresponding author and request for reprints:**

**Jason Y. Adams, MD MS**

**4150 V Street, Suite 3400**

**Sacramento, CA 95817**

**jyadams@ucdavis.edu**

**916-734-3565**

**Contributors**

**JYA, TKW, LPN, MAJ, and CNC conceived and designed the study. JYA, TKW, LPN, MAJ, NTP, ASG, MRL, FR, CG and DB performed data collection and curation. All authors participated in analysis and interpretation of study data. CG, DB and JYA wrote the first draft of the manuscript and all authors participated in revision of the manuscript for important intellectual content. JYA, TKW, LPN, CNC, and MAJ obtained funding for the study, and JYA and CNC provided overall study supervision. JYA, TKW, LPN, MAJ, and CNC had full access to the data used in the study and take full responsibility for the integrity of the data and the accuracy of the data analysis. JYA was responsible for the final decision to submit.**

**Conflicts of Interest and Source of Funding**

**Funding for this study was provided by the US Army Medical Research and Development Command, Award Number W81XWH-18-0072. The content is solely the responsibility of the authors and does not necessarily represent the official views of the United States Department of Defense. The funders had no role in study design, analysis or interpretation, writing of the manuscript, or the decision to submit for publication. MAJ, TKW, LPN, and JYA are co-founders of Certus Critical Care Inc., a company working to translate aspects of the current work to the bedside. The remaining authors have disclosed that they do not have any conflicts of interest.**

**Keywords: critical care, machine learning, cardiac output, fluid therapy, hemodynamics, swine**

**Supplemental digital content is available for this article.**

**
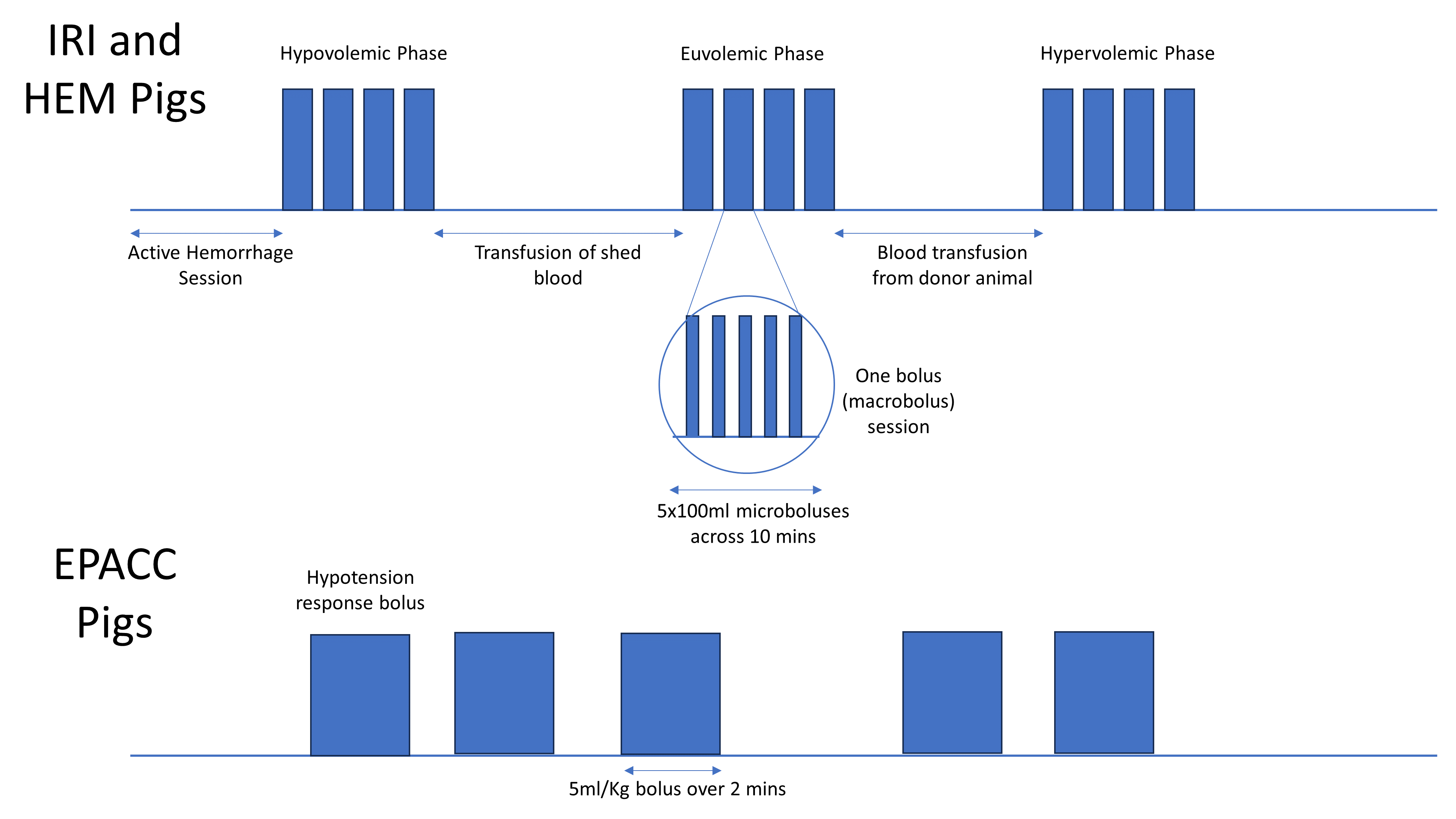
**

**Supplementary Figure 1: High-Level Overview of Study Resuscitation Strategies.** Hemorrhagic shock (HEM) and ischemia reperfusion injury (IRI) animals both underwent acute hemorrhage and IRI animals also underwent 20 minutes of endovascular aortic occlusion at the end of the hemorrhage period. HEM and IRI animals then received four sequential 500 ml crystalloid boluses during each of three experimental physiologic phases (hypovolemic, euvolemic, and hypervolemic) to enable assessment of response to fluid boluses across a broad range of cardiac filling pressures. Animals receiving endovascular perfusion augmentation for critical care (EPACC) received intermittent hypotension-triggered, weight-based fluid boluses after hemorrhage and endovascular aortic occlusion (variable number of boluses per animal over a four hour period).

**Supplementary Methods**

**Animal Models and Data Collection**

**Anesthesia and Prep:**

Yorkshire swine weighing >55kg were allowed to acclimatize to their enclosures for at least 48 hours. Animals were fasted overnight prior to the study date but were allowed free access to water. General anesthesia was induced with Telazol, 5.0-7.0 mg/kg IM, and supplemented with inhaled isoflurane by mask, followed by intubation and placement of a temperature probe, end-tidal carbon dioxide monitor, EKG leads, and oxygen saturation monitor. Animals were placed supine for surgical instrumentation and maintained with ~2% isoflurane and 100% oxygen at 2 L/min mixed with room air at 1.5 L/min. Normal body temperature was maintained using an underbody warmer. Vascular access was similar in all animals. The external jugular vein was cannulated for maintenance intravenous (IV) fluid administration, vasopressor administration, and central venous pressure. A 5-7 Fr sheath was placed in the right common femoral artery and the left axillary artery to measure and record invasive blood pressures. Standard fluid columns were placed at the phlebostatic axis and zeroed to atmospheric pressure prior to collecting invasive blood pressure data. The right brachial artery was accessed with a 5 Fr micropuncture sheath for blood draws. Lastly, the left femoral vein was accessed using a 9 Fr MAC catheter for hemorrhage, blood transfusion, and fluid resuscitation. Additional vascular instrumentation specific to each cohort are described in the following sections.

***Ischemia-Reperfusion Injury with Intermittent Occlusion of the Supraceliac Aorta (EPACC) Model***

**Ventilation and Surgical Instrumentation:**

Mechanical ventilation support was set to pressure control adjusted for an initial tidal volume of 8 mL/kg and PEEP of 5 cmH2O was provided and was adjusted to a goal end-tidal CO2 of 40 ± 5 mmHg using a lung protective strategy (use of the ARDSnet low PEEP-FiO2 table and goal tidal volumes <= 8 ml/kg). Following IV access, VetivexTM pHyLyteTM solution (referred to here as plasmalyte) was administered as a 1L IV initial bolus then 10 mL/kg/hr IV infusion through end of instrumentation, at which time the rate was reduced to 5 mL/kg/hr for the remainder of the experiment. An IV norepinephrine infusion was titrated to maintain a goal mean arterial pressure (MAP) ≥ 60 mmHg during surgical instrumentation.

In addition to vascular access described in the previous section, the left femoral artery was accessed and sheath inserted for aortic balloon catheter placement into the descending aorta (Zone 1). Following laparotomy and cystostomy tube placement, a splenectomy was performed to minimize hemodynamic variation from autotransfusion. Ultrasound flow probes were placed around the supraceliac aorta, left carotid artery, and left renal artery.

**Injury and Resuscitation Phases:**

Animals underwent a controlled 30% total body blood volume hemorrhage over 30 minutes (T0-T30). Then the aortic balloon was inflated to complete occlusion from T30-T60. During the full occlusion period, an infusion of calcium gluconate was initiated at T45, then beginning at T55 animals were transfused back to 95% of the shed whole blood volume over 15 minutes. At T60 animals entered the critical care phase and underwent a 15-minute automated balloon weaning protocol using an automated syringe pump to titrate the balloon volume and maintain a proximal MAP of 65 mmHg. At T75, animals were randomized to either a standardized critical care algorithm with automated fluid and vasopressor support only (SCC), or one of 5 groups with continued automated aortic balloon support paired with SCC (EPACC+SCC). Resuscitation in the SCC group involved aortic balloon removal at T75 then implementation of a standardized automated crystalloid bolus and norepinephrine titration protocol through the end of study, T330. Animals randomized to one of the EPACC+SCC arms received similar critical care support with the addition of automated aortic balloon support based on closed-loop adaptive algorithms to control the balloon volume. Balloon support was provided through T150 or T270 (90 minutes and 210 minutes into critical care) then the balloon was removed. Once removed, continued support was provided with crystalloid boluses and norepinephrine titration using the automated SCC algorithm. For all groups, automated protocols were informed by proximal MAP and central venous pressure inputs with an overall treatment target MAP range of 60-70 mmHg.

***Hemorrhagic Shock (HEM) and Ischemia Reperfusion Injury (IRI) Models***

**Ventilation and Surgical Instrumentation:**

Mechanical ventilation support was set to volume control with an initial tidal volume of 8 mL/kg and PEEP of 5 cmH2O was provided and was adjusted to a goal end-tidal CO2 of 40 ± 5 mmHg using a lung protective strategy (use of the ARDSnet low PEEP-FiO2 table and goal tidal volumes <= 8 ml/kg). Intraoperative IV fluids were administered at 5 mL/kg/hr once venous access was obtained. A continuous infusion of norepinephrine (starting dose 0.02 mcg/kg/min, titrated until the goal MAP of 60-70 mm Hg was achieved) was used through the end of surgical setup, then the rate was held constant once the timed portion of the study began. Additional vascular instrumentation included the right external jugular vein, accessed with a 7-9 Fr sheath catheter for pulmonary artery catheter placement, and the right carotid artery, accessed for placement of the pressure-volume (PV) loop catheter into the left ventricle (PV loop insertion and measurement detail provided at the end of this section). A laparotomy was performed to facilitate Foley catheter placement via cystotomy. In the IRI animals, a splenectomy was performed to prevent autotransfusion, a flow probe was placed on the supraceliac aorta, and an aortic balloon catheter was placed through a left femoral arterial sheath and into the descending aorta (Zone 1).

**Injury and Resuscitation Phases:**

*Hypovolemic Phase*: At time 0 (T0), HEM animals were subjected to a controlled hemorrhage of 25% of their total blood volume (calculated as weight (kg) x 0.6) over 30 minutes. Animals in the IRI group were subjected to a controlled hemorrhage of 20% total blood volume over 30 minutes, followed by 10 minutes of hypotension, 20 minutes of partial or full aortic occlusion, and a 5 minute balloon wean phase (4 minutes of balloon wean then balloon removal during that last minute of the phase). Following hemorrhage ± partial or full aortic occlusion, all animals underwent a period of fluid bolus administration over a one-hour time period. Specifically, animals received 500 mL “macroboluses” of plasmalyte delivered as five sequential “microboluses” at a rate of 100 mL/min every other minute for 10 minutes followed by a 5-minute lockout period with only data collection. This was repeated 4 times for a total of 2 liters of fluid over one hour. Ventilator settings were not adjusted during the fluid bolus administration periods.

*Euvolemic Phase*: Following the 2 liters of plasmalyte in the hypovolemic phase, animals were resuscitated with their shed blood to return them to a normovolemic state with the goal that some animals would be fluid responsive and some non-responsive. Following blood resuscitation, animals received another 2 liters of plasmalyte in the same fashion as in the hypovolemic phase.

*Hypervolemic Phase*: After the second 2 liters of plasmalyte in the euvolemic phase, animals were transfused from donor animals to create a hypervolemic state with the goal that most animals would no longer be fluid responsive. The HEM animals were given a homologous blood transfusion from donor animals of 25% of estimated blood volume over 30 minutes. Animals in the IRI group were given a 30% homologous blood transfusion over 30 minutes. Following the blood transfusion, the animals were given 2 liters of plasmalyte as described earlier. A diphenhydramine dose of 1 mg/kg IV was given for transfusion reactions as indicated by urticaria, blotchy skin, or pulmonary hypertension. At the end of the final 2-liter resuscitation period, animals were sacrificed using intravenous ethanol (1 ml/10 pounds of body weight) while under general anesthesia and underwent necropsy.

*Physiologic Measurements:* During all phases, physiologic parameters were collected in real time using a PowerLab data collection system. Parameters measured included heart rate, blood pressure, central venous pressure, core temperature, pulse oxygenation, cardiac output measured with the PV loop catheter, FiO2, and end-tidal carbon dioxide concentration. Ventilation waveforms were obtained using a pneumotachometer connected to the PowerLab data collection system.

**PV-loop catheter insertion and measurements:**

1. Catheter Insertion:

- A specialized catheter was inserted under fluoroscopic guidance through the carotid artery access, employing a 9 Fr introducer sheath. This allowed precise placement of the catheter within the left ventricle.

1. Catheter Placement:
   - The catheter was meticulously advanced through the carotid artery, ensuring a secure and minimally invasive pathway via the 9 Fr introducer sheath.
2. Calibration:
   - After the catheter was properly positioned within the left ventricle, calibration was performed to ensure the accuracy of pressure and volume measurements. This step involved establishing a zero-reference point for pressure measurements to account for variations due to factors such as altitude or instrument-specific characteristics.
3. Pressure Measurement:
   - The catheter, once in place, continuously measured the pressure within the left ventricle, providing valuable data on pressure variations throughout the cardiac cycle, including systole and diastole.
4. Volume Measurement:
   - The catheter's sensor detected changes in the dimensions of the left ventricle, capturing data on volume changes during the cardiac cycle. This information was used to generate PV loops, representing the relationship between pressure and volume.
5. Monitoring:
   - Real-time monitoring of the PV loop data was conducted using specialized equipment and software. This allowed for the assessment of cardiac performance under various conditions and interventions, with a particular focus on the shape and parameters of the PV loop.

**
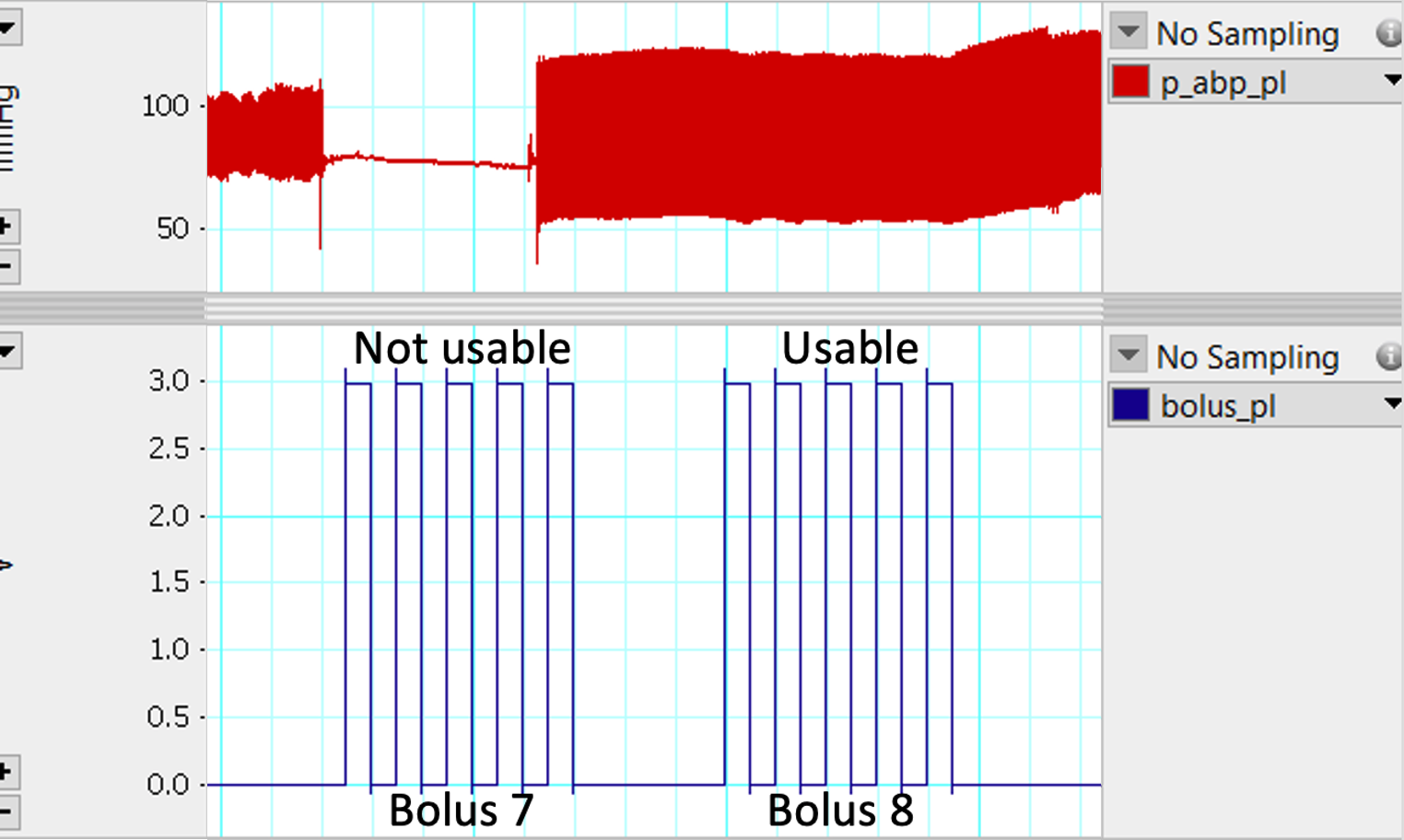
**

**Supplementary Figure 2: Signal dampening in arterial blood pressure tracing.** Example of arterial blood pressure tracing (red tracing) at low magnification with severe signal dampening affecting of the 1st 500 ml bolus (bottom purple tracing shows a voltage signal from the infusion pump used to delineate when each of five 100 ml microboluses were administered). Because the signal dampening was not addressed and corrected prior to the start of the bolus, the bolus was excluded from training/testing datasets.


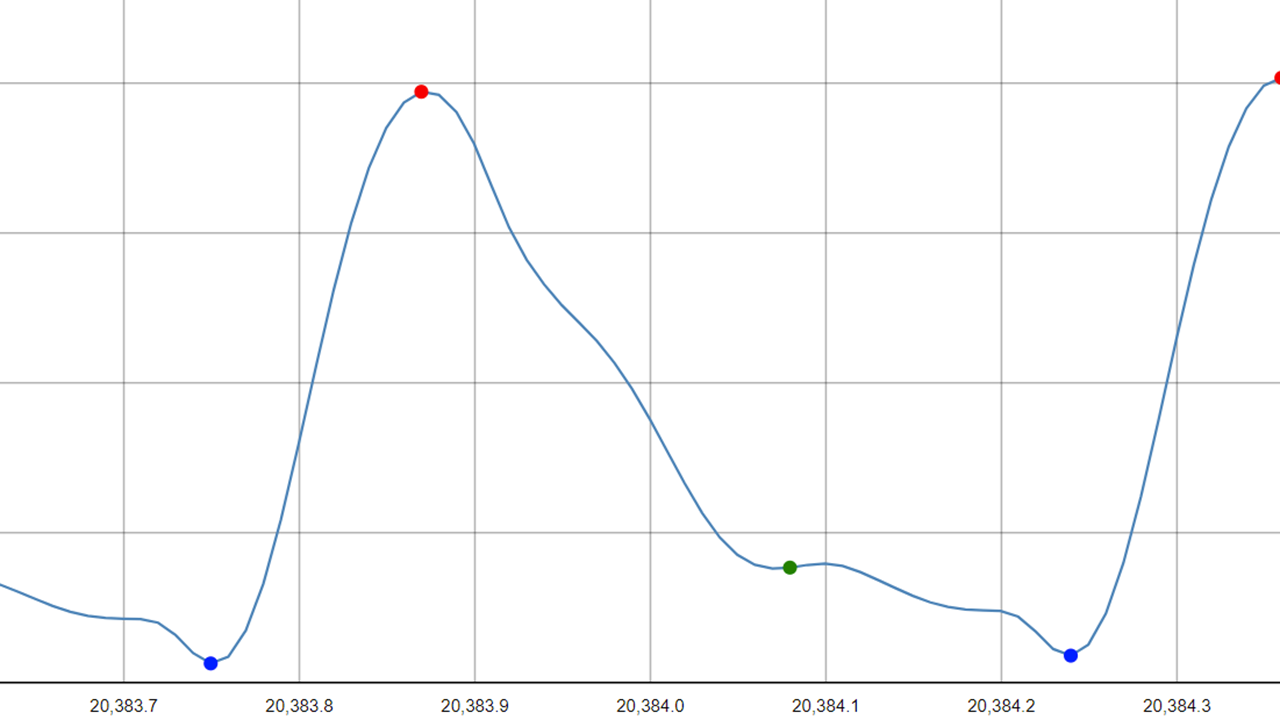


**Supplementary Figure 3: Arterial pressure waveform with core feature markers** for systolic blood pressure (red), diastolic blood pressure (blue), and the dicrotic notch (green). Rule-based algorithms created for core feature detection also allowed calculation of the additional expert-informed input features in Supplemental Table 3.

| **Supplementary Table 1:** Classification performance of random forest model (using statistical feature selection and correlation threshold of 0.9) on datasets separated by injury model | | | | | | | |
| --- | --- | --- | --- | --- | --- | --- | --- |
| **Trained On** | **Tested On** | **Accuracy** | **AUROC** | **Precision** | **Recall** | **Specificity** | **AUPRC** |
| HEM | IRI | 0.81 | 0.84 | 0.76 | 0.85 | 0.77 | 0.79 |
|  | EPACC | 0.82 | 0.66 | 0.81 | 1.00 | 0.19 | 0.85 |
| EPACC | HEM | 0.67 | 0.76 | 0.67 | 0.60 | 0.73 | 0.71 |
|  | IRI | 0.71 | 0.85 | 0.63 | 0.91 | 0.53 | 0.75 |
| IRI | HEM | 0.63 | 0.76 | 1.00 | 0.21 | 1.00 | 0.77 |
|  | EPACC | 0.67 | 0.81 | 0.94 | 0.61 | 0.87 | 0.92 |

IRI, Ischemia-Reperfusion Injury; HEM, Hemorrhagic shock; EPACC, Ischemia-reperfusion injury followed by intermittent occlusion of the supraceliac aorta; AUROC, Area under receiver operating characteristic curve; AUPRC, Area under precision recall curve

| **Supplementary Table 2:** Description of the composition of the 29 pig level splits of the entire dataset by bolus number, proportion fluid responsive, and proportion in each injury model. | | | | | | | | | | |
| --- | --- | --- | --- | --- | --- | --- | --- | --- | --- | --- |
| **Split** | **Training (n)** | **Test (n)** | **% FR, Training** | **% FR, Test** | **% IRI, Training** | **% EPACC, Training** | **% HEM, Training** | **% IRI, Test** | **% EPACC, Test** | **% HEM, Test** |
| 1 | 276 | 118 | 55 | 64 | 34 | 29 | 37 | 21 | 52 | 27 |
| 2 | 268 | 126 | 59 | 56 | 35 | 32 | 32 | 19 | 43 | 38 |
| 3 | 283 | 111 | 60 | 53 | 32 | 33 | 35 | 25 | 42 | 32 |
| 4 | 279 | 115 | 61 | 50 | 30 | 37 | 33 | 30 | 33 | 37 |
| 5 | 252 | 142 | 58 | 57 | 27 | 37 | 36 | 35 | 34 | 31 |
| 6 | 260 | 134 | 54 | 65 | 27 | 38 | 34 | 36 | 31 | 34 |
| 7 | 265 | 129 | 58 | 57 | 31 | 32 | 37 | 29 | 43 | 28 |
| 8 | 261 | 133 | 55 | 63 | 18 | 35 | 47 | 54 | 38 | 8 |
| 9 | 297 | 97 | 57 | 62 | 33 | 33 | 34 | 22 | 45 | 33 |
| 10 | 250 | 144 | 55 | 62 | 25 | 36 | 38 | 39 | 35 | 26 |
| 11 | 267 | 127 | 58 | 57 | 32 | 38 | 30 | 27 | 31 | 43 |
| 12 | 262 | 132 | 61 | 51 | 24 | 41 | 35 | 42 | 25 | 33 |
| 13 | 259 | 135 | 61 | 52 | 19 | 34 | 47 | 51 | 39 | 10 |
| 14 | 292 | 102 | 55 | 65 | 38 | 29 | 32 | 7 | 54 | 39 |
| 15 | 269 | 125 | 57 | 59 | 28 | 34 | 38 | 35 | 40 | 25 |
| 16 | 281 | 113 | 59 | 54 | 35 | 34 | 31 | 18 | 41 | 42 |
| 17 | 263 | 131 | 56 | 61 | 27 | 38 | 35 | 37 | 32 | 31 |
| 18 | 283 | 111 | 58 | 59 | 34 | 32 | 34 | 21 | 46 | 33 |
| 19 | 274 | 120 | 58 | 57 | 35 | 33 | 32 | 18 | 42 | 39 |
| 20 | 262 | 132 | 61 | 52 | 29 | 37 | 33 | 32 | 33 | 36 |
| 21 | 273 | 121 | 57 | 60 | 36 | 31 | 33 | 18 | 46 | 36 |
| 22 | 289 | 105 | 56 | 62 | 25 | 29 | 46 | 44 | 55 | 1 |
| 23 | 241 | 153 | 60 | 54 | 29 | 38 | 33 | 32 | 33 | 35 |
| 24 | 246 | 148 | 57 | 59 | 35 | 42 | 23 | 23 | 25 | 52 |
| 25 | 272 | 122 | 57 | 59 | 34 | 28 | 38 | 21 | 52 | 26 |
| 26 | 252 | 142 | 56 | 61 | 23 | 33 | 45 | 44 | 42 | 15 |
| 27 | 297 | 97 | 56 | 63 | 29 | 29 | 42 | 34 | 56 | 10 |
| 28 | 279 | 115 | 61 | 50 | 29 | 39 | 32 | 34 | 27 | 39 |
| 29 | 253 | 141 | 60 | 55 | 33 | 37 | 30 | 25 | 33 | 42 |

FR, Fluid responsive; IRI, Ischemia-Reperfusion Injury; HEM, Hemorrhagic shock; EPACC, Ischemia-reperfusion injury followed by intermittent occlusion of the supraceliac aorta

| **Supplementary Table 3:** Feature description table | |
| --- | --- |
| **Feature Code** | **Feature Description** |
| median_sys_pres | Median of systolic pressure |
| std_sys_pres | Standard deviation of systolic pressure |
| median_dias_pres | Median of diastolic pressure |
| std_dias_pres | Standard deviation of diastolic pressure |
| median_dic_pres | Median of dicrotic notch pressure |
| std_dic_pres | Standard deviation of dicrotic notch pressure |
| median_map | Median of mean arterial blood pressure |
| std_map | Standard deviation of mean arterial blood pressure |
| median_heart_rate | Median of heart rate |
| std_heart_rate | Standard deviation of heart rate |
| median_t_sys_rise | Median of duration of the systolic rise phase (beat start to systolic maximum) |
| std_t_sys_rise | Standard deviation of duration of the systolic rise phase (beat start to systolic maximum) |
| median_t_sys | Median of duration of the systolic phase (beat start to dicrotic notch) |
| std_t_sys | Standard deviation of duration of the systolic phase (beat start to dicrotic notch) |
| median_t_dia | Median of duration of the diastolic phase (dicrotic notch to beat end) |
| std_t_dia | Standard deviation of duration of the diastolic phase (dicrotic notch to beat end) |
| median_ibi | Median of inter-beat interval |
| std_ibi | Standard deviation of inter-beat interval |
| median_pulse_pres | Median of pulse pressure |
| std_pulse_pres | Standard deviation of pulse pressure |
| median_avg_sys_rise | Median of average of the systolic rise portion of the waveform |
| std_avg_sys_rise | Standard deviation of average of the systolic rise portion of the waveform |
| median_avg_sys | Median of average of the systolic portion of the waveform |
| std_avg_sys | Standard deviation of average of the systolic portion of the waveform |
| median_avg_dia | Median of average of the of the diastolic portion of the waveform |
| std_avg_dia | Standard deviation of average of the of the diastolic portion of the waveform |
| median_pp_area | Median of area under the beat waveform |
| std_pp_area | Standard deviation of area under the beat waveform |
| median_pp_area_nor | Median of area under the beat normalized by the number of samples |
| std_pp_area_nor | Standard deviation of area under the beat normalized by the number of samples |
| median_sys_area | Median of area under the systolic phase of the beat (beat start to dicrotic notch) |
| std_sys_area | Standard deviation of area under the systolic phase of the beat (beat start to dicrotic notch) |
| median_sys_area_nor | Median of area under the systolic phase normalized by the number of samples |
| std_sys_area_nor | Standard deviation of area under the systolic phase normalized by the number of samples |
| median_sys_rise_area | Median of area from the start of the beat to the systolic maximum |
| std_sys_rise_area | Standard deviation of area from the start of the beat to the systolic maximum |
| median_sys_rise_area_nor | Median of area from the start of the beat to the systolic maximum normalized by the number of samples |
| std_sys_rise_area_nor | Standard deviation of area from the start of the beat to the systolic maximum normalized by the number of samples |
| median_sys_dec_area | Median of area from the systolic maximum to the dicrotic notch |
| std_sys_dec_area | Standard deviation of area from the systolic maximum to the dicrotic notch |
| median_sys_dec_area_nor | Median of area from the systolic maximum to the dicrotic notch normalized by the number of samples |
| std_sys_dec_area_nor | Standard deviation of area from the systolic maximum to the dicrotic notch normalized by the number of samples |
| median_dec_area | Median of area from the systolic maximum to the start of the next beat |
| std_dec_area | Standard deviation of area from the systolic maximum to the start of the next beat |
| median_dec_area_nor | Median of area from the systolic maximum to the start of the next beat normalized by the number of samples |
| std_dec_area_nor | Standard deviation of area from the systolic maximum to the start of the next beat normalized by the number of samples |
| median_dia_area | Median of area under the diastolic portion of the waveform |
| std_dia_area | Standard deviation of area under the diastolic portion of the waveform |
| median_dia_area_nor | Median of area under the diastolic portion of the waveform normalized by the number of samples |
| std_dia_area_nor | Standard deviation of area under the diastolic portion of the waveform normalized by the number of samples |

| **Supplementary Table 4:** Split-wise comparison of classification performance of random forest algorithm with different feature selection methods and PPV based classification for holdout sets | | | | | | | |
| --- | --- | --- | --- | --- | --- | --- | --- |
| **Feature Selection Method** | **Split** | **Classification performance of Random Forest** | | | **Classification Performance of PPV** | | |
|  |  | **AUROC** | **Recall** | **Specificity** | **AUROC** | **Recall** | **Specificity** |
| Statistical Feature Selection | 1 | 0.79 | 0.92 | 0.42 | 0.76 | 0.89 | 0.55 |
|  | 2 | 0.89 | 0.92 | 0.73 | 0.71 | 0.94 | 0.38 |
|  | 3 | 0.85 | 0.93 | 0.54 | 0.74 | 0.91 | 0.42 |
|  | 4 | 0.82 | 0.96 | 0.5 | 0.78 | 0.92 | 0.53 |
|  | 5 | 0.83 | 0.9 | 0.59 | 0.75 | 0.9 | 0.46 |
|  | 6 | 0.80 | 0.77 | 0.79 | 0.77 | 0.93 | 0.52 |
|  | 7 | 0.85 | 0.82 | 0.71 | 0.73 | 0.92 | 0.47 |
|  | 8 | 0.91 | 0.92 | 0.71 | 0.73 | 0.89 | 0.5 |
|  | 9 | 0.71 | 0.77 | 0.54 | 0.77 | 0.91 | 0.48 |
|  | 10 | 0.78 | 0.78 | 0.65 | 0.75 | 0.88 | 0.51 |
|  | 11 | 0.84 | 0.9 | 0.58 | 0.74 | 0.89 | 0.45 |
|  | 12 | 0.78 | 0.79 | 0.66 | 0.72 | 0.92 | 0.44 |
|  | 13 | 0.86 | 0.89 | 0.65 | 0.73 | 0.92 | 0.48 |
|  | 14 | 0.77 | 0.76 | 0.64 | 0.74 | 0.94 | 0.45 |
|  | 15 | 0.84 | 0.85 | 0.63 | 0.74 | 0.92 | 0.51 |
|  | 16 | 0.89 | 0.89 | 0.77 | 0.73 | 0.93 | 0.42 |
|  | 17 | 0.86 | 0.83 | 0.71 | 0.74 | 0.92 | 0.47 |
|  | 18 | 0.85 | 0.82 | 0.7 | 0.73 | 0.91 | 0.48 |
|  | 19 | 0.84 | 0.94 | 0.52 | 0.75 | 0.9 | 0.46 |
|  | 20 | 0.86 | 0.94 | 0.52 | 0.72 | 0.9 | 0.47 |
|  | 21 | 0.86 | 0.72 | 0.84 | 0.74 | 0.95 | 0.4 |
|  | 22 | 0.87 | 0.98 | 0.53 | 0.75 | 0.9 | 0.5 |
|  | 23 | 0.82 | 0.87 | 0.57 | 0.73 | 0.89 | 0.47 |
|  | 24 | 0.79 | 0.81 | 0.63 | 0.79 | 0.91 | 0.53 |
|  | 25 | 0.86 | 0.89 | 0.7 | 0.77 | 0.91 | 0.46 |
|  | 26 | 0.87 | 0.87 | 0.63 | 0.73 | 0.88 | 0.53 |
|  | 27 | 0.89 | 0.87 | 0.69 | 0.72 | 0.88 | 0.46 |
|  | 28 | 0.85 | 0.81 | 0.68 | 0.75 | 0.91 | 0.46 |
|  | 29 | 0.86 | 0.9 | 0.66 | 0.73 | 0.93 | 0.4 |
| Mutual Information | 1 | 0.74 | 0.92 | 0.4 | 0.76 | 0.89 | 0.55 |
|  | 2 | 0.85 | 0.89 | 0.8 | 0.71 | 0.94 | 0.38 |
|  | 3 | 0.85 | 0.95 | 0.52 | 0.74 | 0.91 | 0.42 |
|  | 4 | 0.79 | 0.95 | 0.43 | 0.78 | 0.92 | 0.53 |
|  | 5 | 0.84 | 0.91 | 0.57 | 0.75 | 0.9 | 0.46 |
|  | 6 | 0.80 | 0.8 | 0.74 | 0.77 | 0.93 | 0.52 |
|  | 7 | 0.84 | 0.79 | 0.71 | 0.73 | 0.92 | 0.47 |
|  | 8 | 0.88 | 0.92 | 0.69 | 0.73 | 0.89 | 0.5 |
|  | 9 | 0.70 | 0.77 | 0.65 | 0.77 | 0.91 | 0.48 |
|  | 10 | 0.75 | 0.78 | 0.61 | 0.75 | 0.88 | 0.51 |
|  | 11 | 0.83 | 0.89 | 0.55 | 0.74 | 0.89 | 0.45 |
|  | 12 | 0.79 | 0.76 | 0.69 | 0.72 | 0.92 | 0.44 |
|  | 13 | 0.87 | 0.91 | 0.55 | 0.73 | 0.92 | 0.48 |
|  | 14 | 0.76 | 0.74 | 0.64 | 0.74 | 0.94 | 0.45 |
|  | 15 | 0.85 | 0.84 | 0.65 | 0.74 | 0.92 | 0.51 |
|  | 16 | 0.86 | 0.87 | 0.77 | 0.73 | 0.93 | 0.42 |
|  | 17 | 0.83 | 0.83 | 0.73 | 0.74 | 0.92 | 0.47 |
|  | 18 | 0.83 | 0.82 | 0.7 | 0.73 | 0.91 | 0.48 |
|  | 19 | 0.81 | 0.93 | 0.46 | 0.75 | 0.9 | 0.46 |
|  | 20 | 0.87 | 0.96 | 0.5 | 0.72 | 0.9 | 0.47 |
|  | 21 | 0.84 | 0.71 | 0.86 | 0.74 | 0.95 | 0.4 |
|  | 22 | 0.87 | 0.98 | 0.4 | 0.75 | 0.9 | 0.5 |
|  | 23 | 0.79 | 0.87 | 0.53 | 0.73 | 0.89 | 0.47 |
|  | 24 | 0.77 | 0.84 | 0.53 | 0.79 | 0.91 | 0.53 |
|  | 25 | 0.84 | 0.85 | 0.72 | 0.77 | 0.91 | 0.46 |
|  | 26 | 0.86 | 0.88 | 0.61 | 0.73 | 0.88 | 0.53 |
|  | 27 | 0.87 | 0.87 | 0.61 | 0.72 | 0.88 | 0.46 |
|  | 28 | 0.81 | 0.74 | 0.72 | 0.75 | 0.91 | 0.46 |
|  | 29 | 0.84 | 0.9 | 0.66 | 0.73 | 0.93 | 0.4 |
| Permutation Importance | 1 | 0.76 | 0.96 | 0.37 | 0.76 | 0.89 | 0.55 |
|  | 2 | 0.90 | 0.86 | 0.76 | 0.71 | 0.94 | 0.38 |
|  | 3 | 0.86 | 0.93 | 0.52 | 0.74 | 0.91 | 0.42 |
|  | 4 | 0.83 | 1.0 | 0.43 | 0.78 | 0.92 | 0.53 |
|  | 5 | 0.84 | 0.9 | 0.57 | 0.75 | 0.9 | 0.46 |
|  | 6 | 0.80 | 0.79 | 0.77 | 0.77 | 0.93 | 0.52 |
|  | 7 | 0.84 | 0.79 | 0.73 | 0.73 | 0.92 | 0.47 |
|  | 8 | 0.89 | 0.89 | 0.57 | 0.73 | 0.89 | 0.5 |
|  | 9 | 0.65 | 0.73 | 0.46 | 0.77 | 0.91 | 0.48 |
|  | 10 | 0.78 | 0.83 | 0.57 | 0.75 | 0.88 | 0.51 |
|  | 11 | 0.78 | 0.81 | 0.51 | 0.74 | 0.89 | 0.45 |
|  | 12 | 0.76 | 0.76 | 0.55 | 0.72 | 0.92 | 0.44 |
|  | 13 | 0.85 | 0.89 | 0.66 | 0.73 | 0.92 | 0.48 |
|  | 14 | 0.76 | 0.76 | 0.67 | 0.74 | 0.94 | 0.45 |
|  | 15 | 0.79 | 0.86 | 0.49 | 0.74 | 0.92 | 0.51 |
|  | 16 | 0.89 | 0.89 | 0.75 | 0.73 | 0.93 | 0.42 |
|  | 17 | 0.84 | 0.86 | 0.71 | 0.74 | 0.92 | 0.47 |
|  | 18 | 0.81 | 0.85 | 0.57 | 0.73 | 0.91 | 0.48 |
|  | 19 | 0.83 | 0.93 | 0.42 | 0.75 | 0.9 | 0.46 |
|  | 20 | 0.87 | 0.94 | 0.53 | 0.72 | 0.9 | 0.47 |
|  | 21 | 0.85 | 0.71 | 0.82 | 0.74 | 0.95 | 0.4 |
|  | 22 | 0.86 | 0.97 | 0.5 | 0.75 | 0.9 | 0.5 |
|  | 23 | 0.77 | 0.81 | 0.59 | 0.73 | 0.89 | 0.47 |
|  | 24 | 0.77 | 0.81 | 0.53 | 0.79 | 0.91 | 0.53 |
|  | 25 | 0.86 | 0.85 | 0.68 | 0.77 | 0.91 | 0.46 |
|  | 26 | 0.88 | 0.88 | 0.63 | 0.73 | 0.88 | 0.53 |
|  | 27 | 0.86 | 0.87 | 0.64 | 0.72 | 0.88 | 0.46 |
|  | 28 | 0.82 | 0.81 | 0.7 | 0.75 | 0.91 | 0.46 |
|  | 29 | 0.84 | 0.88 | 0.63 | 0.73 | 0.93 | 0.4 |
| Recursive Feature Elimination | 1 | 0.74 | 0.92 | 0.42 | 0.76 | 0.89 | 0.55 |
|  | 2 | 0.88 | 0.87 | 0.76 | 0.71 | 0.94 | 0.38 |
|  | 3 | 0.85 | 0.95 | 0.5 | 0.74 | 0.91 | 0.42 |
|  | 4 | 0.79 | 0.93 | 0.5 | 0.78 | 0.92 | 0.53 |
|  | 5 | 0.83 | 0.9 | 0.56 | 0.75 | 0.9 | 0.46 |
|  | 6 | 0.80 | 0.8 | 0.72 | 0.77 | 0.93 | 0.52 |
|  | 7 | 0.84 | 0.79 | 0.75 | 0.73 | 0.92 | 0.47 |
|  | 8 | 0.89 | 0.92 | 0.73 | 0.73 | 0.89 | 0.5 |
|  | 9 | 0.70 | 0.77 | 0.59 | 0.77 | 0.91 | 0.48 |
|  | 10 | 0.76 | 0.79 | 0.59 | 0.75 | 0.88 | 0.51 |
|  | 11 | 0.83 | 0.9 | 0.55 | 0.74 | 0.89 | 0.45 |
|  | 12 | 0.81 | 0.81 | 0.68 | 0.72 | 0.92 | 0.44 |
|  | 13 | 0.85 | 0.9 | 0.57 | 0.73 | 0.92 | 0.48 |
|  | 14 | 0.78 | 0.73 | 0.69 | 0.74 | 0.94 | 0.45 |
|  | 15 | 0.85 | 0.85 | 0.65 | 0.74 | 0.92 | 0.51 |
|  | 16 | 0.87 | 0.84 | 0.75 | 0.73 | 0.93 | 0.42 |
|  | 17 | 0.85 | 0.84 | 0.71 | 0.74 | 0.92 | 0.47 |
|  | 18 | 0.83 | 0.83 | 0.65 | 0.73 | 0.91 | 0.48 |
|  | 19 | 0.81 | 0.93 | 0.48 | 0.75 | 0.9 | 0.46 |
|  | 20 | 0.83 | 0.96 | 0.52 | 0.72 | 0.9 | 0.47 |
|  | 21 | 0.85 | 0.71 | 0.86 | 0.74 | 0.95 | 0.4 |
|  | 22 | 0.86 | 0.97 | 0.43 | 0.75 | 0.9 | 0.5 |
|  | 23 | 0.80 | 0.87 | 0.53 | 0.73 | 0.89 | 0.47 |
|  | 24 | 0.76 | 0.81 | 0.53 | 0.79 | 0.91 | 0.53 |
|  | 25 | 0.84 | 0.86 | 0.74 | 0.77 | 0.91 | 0.46 |
|  | 26 | 0.85 | 0.9 | 0.61 | 0.73 | 0.88 | 0.53 |
|  | 27 | 0.86 | 0.87 | 0.58 | 0.72 | 0.88 | 0.46 |
|  | 28 | 0.82 | 0.74 | 0.7 | 0.75 | 0.91 | 0.46 |
|  | 29 | 0.85 | 0.9 | 0.64 | 0.73 | 0.93 | 0.4 |

AUROC, Area under receiver operating characteristic curve; PPV, Pulse Pressure Variation

**
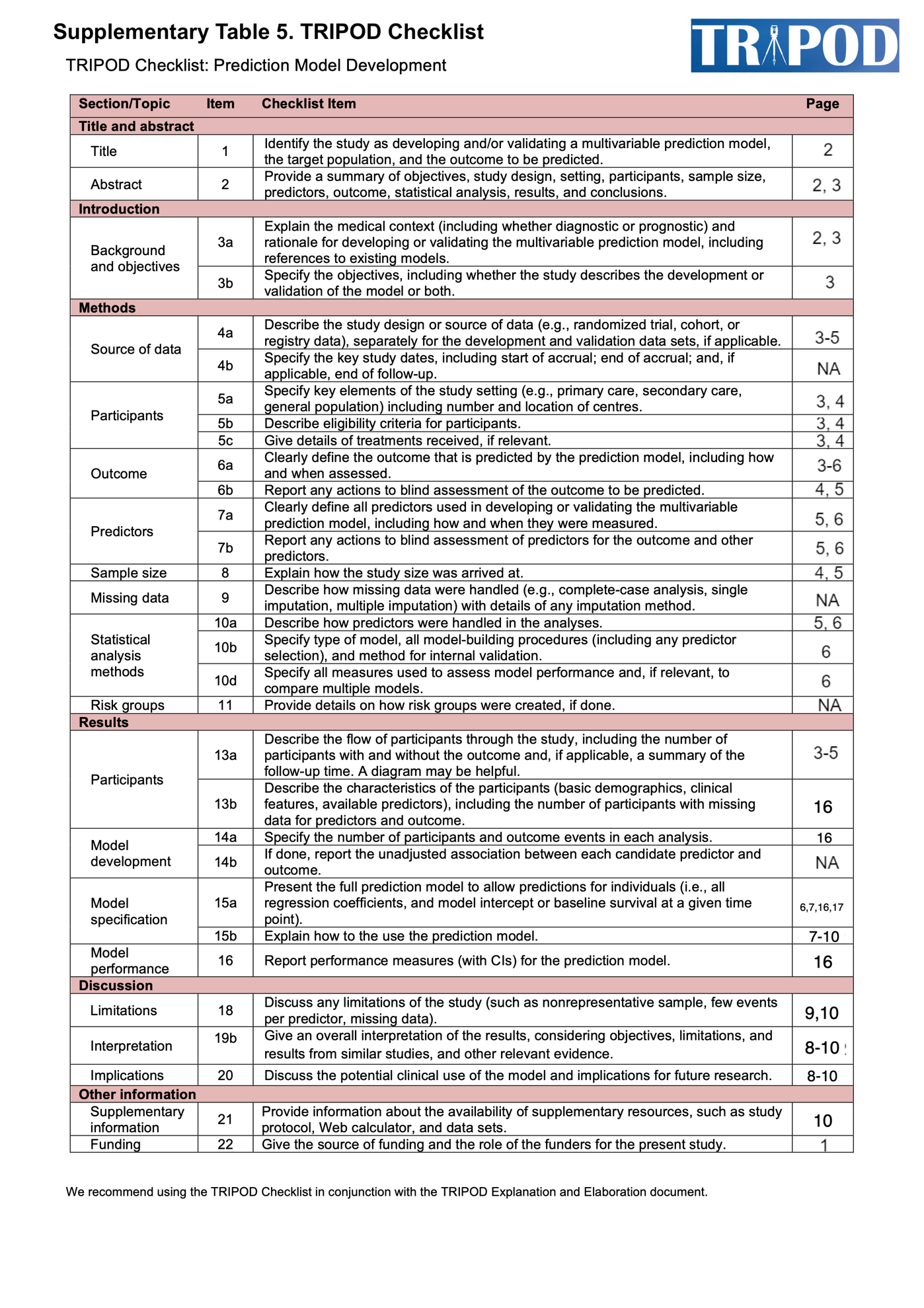
**

*
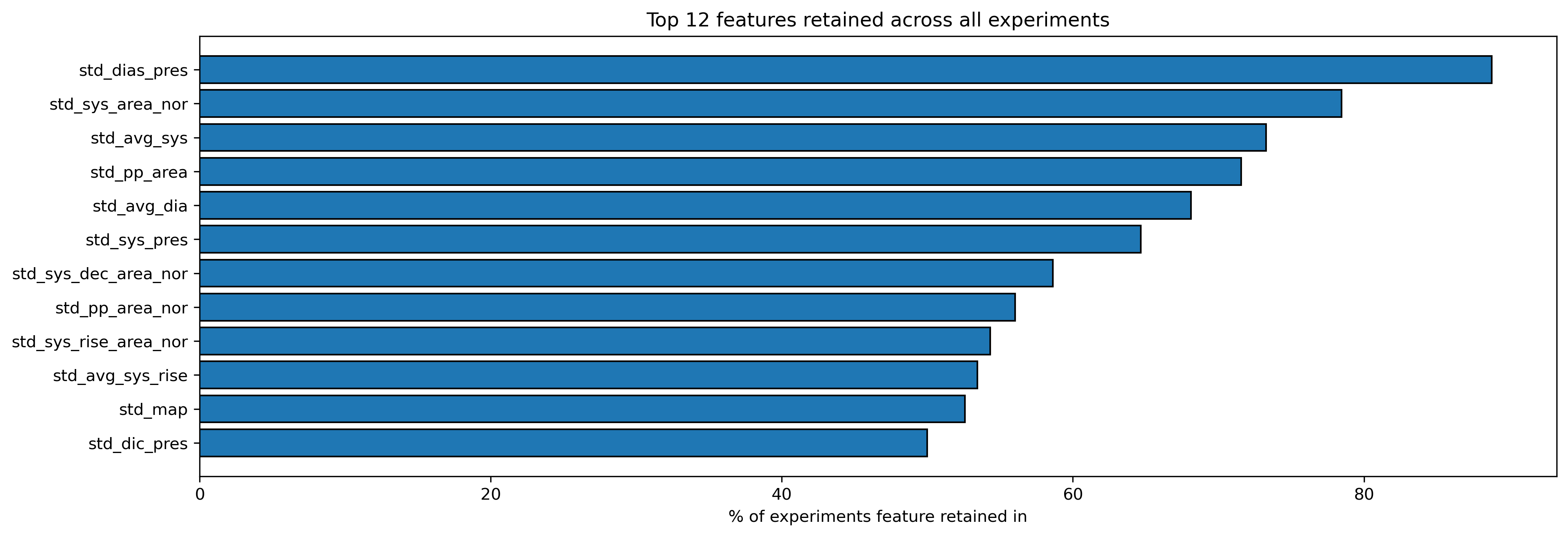
*

**Supplementary Figure 4:** **Most retained features.** List of Features retained by over 50% of 4x29 feature selection experiments.

| **Supplementary Table 6:** Hemodynamic Variables* Before and After Fluid Challenges | | | | |
| --- | --- | --- | --- | --- |
|  | **Fluid Responsive Boluses** | | **Fluid Non-Responsive Boluses** | |
| **Hemodynamic Variable** | **Pre-Bolus** | **Post-Bolus** | **Pre-Bolus** | **Post-Bolus** |
| Heart Rate | 139.9 | 123.2 | 115.8 | 97.6 |
| SBP | 89.6 | 92.6 | 96.8 | 83.8 |
| DBP | 44.4 | 44.2 | 56.5 | 49 |
| MAP | 64 | 66.3 | 75.4 | 65.7 |
| % Change in SV | NA | 35.7 | NA | 5.7 |
| *Values represent the median of all beats measured in the 60 seconds pre- and post-bolus; SBP, systolic blood pressure; DBP, diastolic blood pressure; MAP, mean arterial blood pressure; SV, stroke volume | | | | |

| **Supplementary Table 7:** Comparison of average training performance of different classification algorithms across 29 training splits. Each of them was evaluated with statistical feature selection. | | | | | | |
| --- | --- | --- | --- | --- | --- | --- |
| **Algorithm** | **Accuracy** | **AUROC** | **Precision** | **Recall** | **Specificity** | **AUPRC** |
| SVM | 0.7 | 0.83 | 0.74 | 0.81 | 0.55 | 0.86 |
| LR | 0.74 | 0.80 | 0.78 | 0.79 | 0.68 | 0.84 |
| XGB | 0.75 | 0.86 | 0.84 | 0.72 | 0.80 | 0.88 |
| RF | 0.78 | 0.86 | 0.80 | 0.84 | 0.70 | 0.88 |

AUROC, Area under receiver operating characteristic curve; AUPRC, Area under precision recall curve; SVM, Support Vector Machine; LR, Logistic Regression; XGB, Extreme Gradient Boosting (XGBoost); RF, Random Forest

**
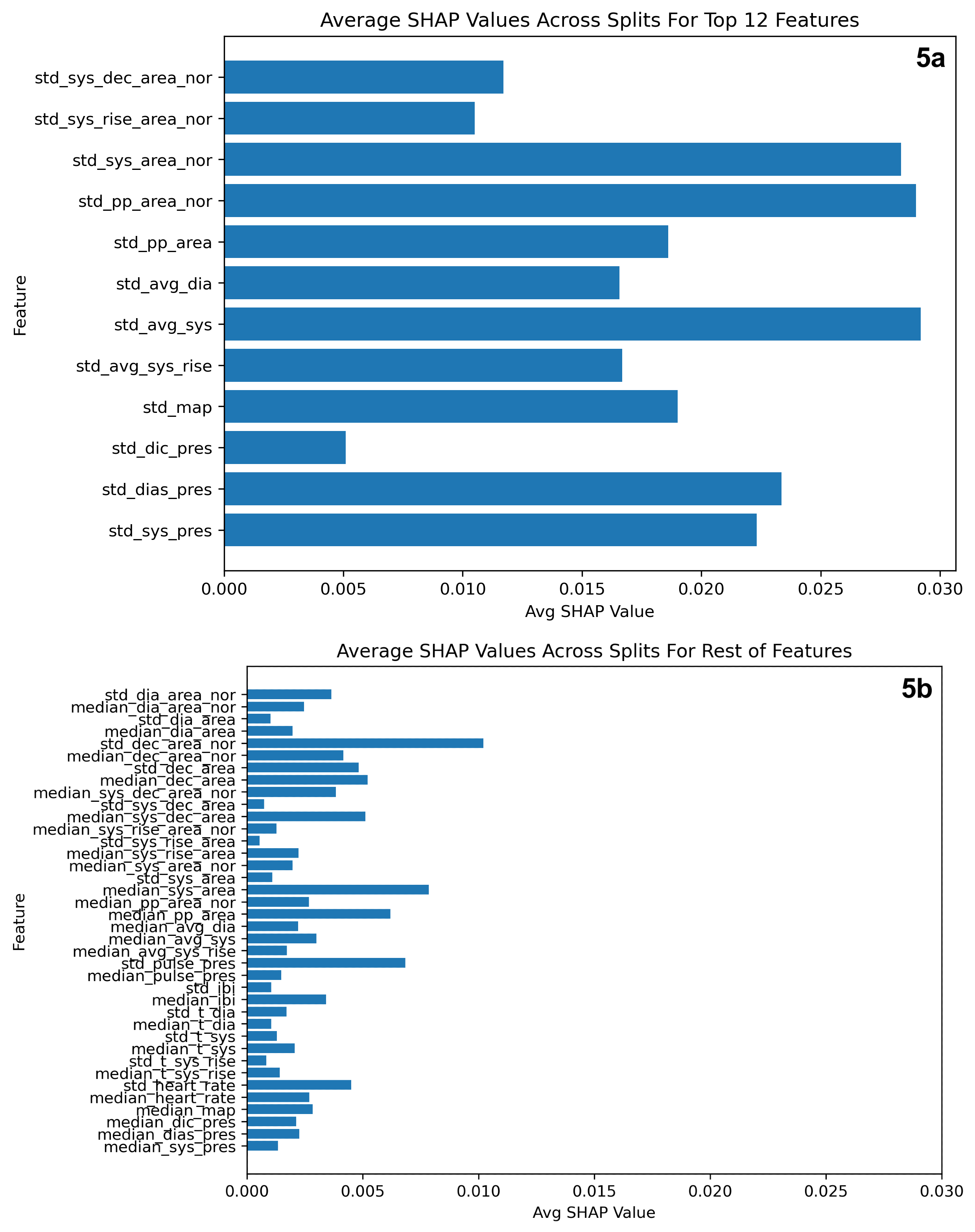
**

**Supplementary Figure 5:** **SHAP values for all features.** SHAP values of all features (**a** includes the 12 features retained in over 50% of 4x29 feature selection experiments, **b** includes scores for remaining features)

| **Supplementary Table 8:** Classification performance metrics on training and holdout sets across all 29 dataset splits for pulse pressure variation (PPV) as a univariate predictor of change in stroke volume in response to bolus administration, and the 12-feature random forest model. | | | | | | | |
| --- | --- | --- | --- | --- | --- | --- | --- |
| **Data** | **Feature Selection Method** | **Accuracy** | **AUROC** | **Precision** | **Recall** | **Specificity** | **AUPRC** |
| Train | PPV | 0.76±0.01 | 0.77±0.02 | 0.74±0.02 | 0.9±0.01 | 0.57±0.03 | 0.76±0.02 |
|  | 12-Feature Model | 0.78±0.05 | 0.85±0.06 | 0.79±0.08 | 0.84±0.08 | 0.69±0.13 | 0.87±0.06 |
| Test | PPV | 0.74±0.02 | 0.73±0.02 | 0.71±0.02 | 0.91±0.01 | 0.49±0.03 | 0.73±0.02 |
|  | 12-Feature Model | 0.76±0.02 | 0.82±0.02 | 0.77±0.02 | 0.86±0.02 | 0.64±0.03 | 0.83±0.02 |

AUROC, Area under receiver operating characteristic curve; AUPRC, Area under precision recall curve; PPV, Pulse pressure variation


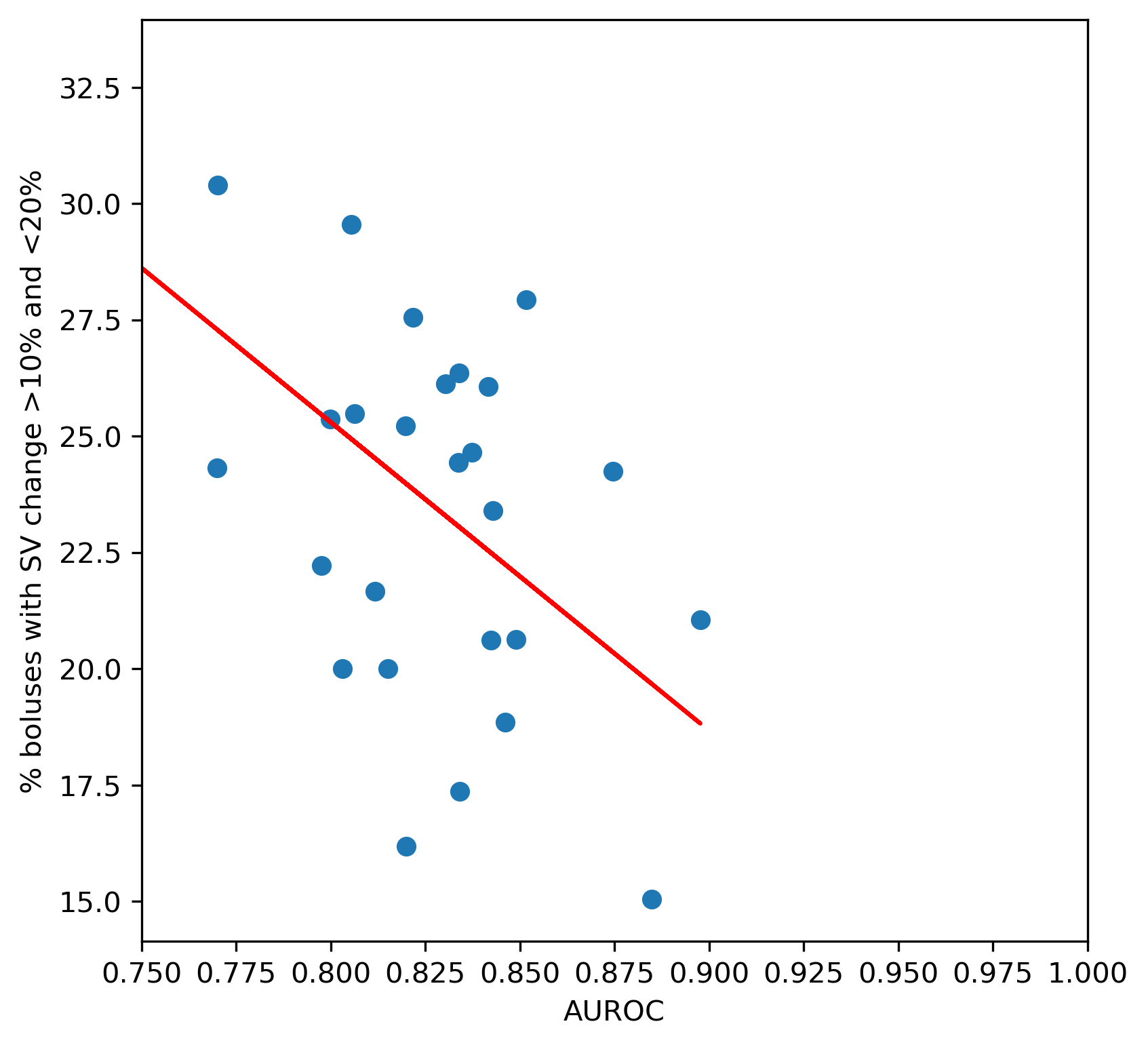


**Supplementary Figure 6: Correlation between the proportion of boluses in the grey zone near the target label decision boundary and machine learning model performance.** The plot shows the relationship between the percentage of boluses in each of the 29 holdout test datasets with a change in stroke volume (SV) between 10-20%, representing a grey zone flanking the 15% boundary used to define fluid responsiveness, and the corresponding model’s area under receiver operating characteristic curve (AUROC). Data are from models trained using the 12-features consistently retained across feature selection methods.

*
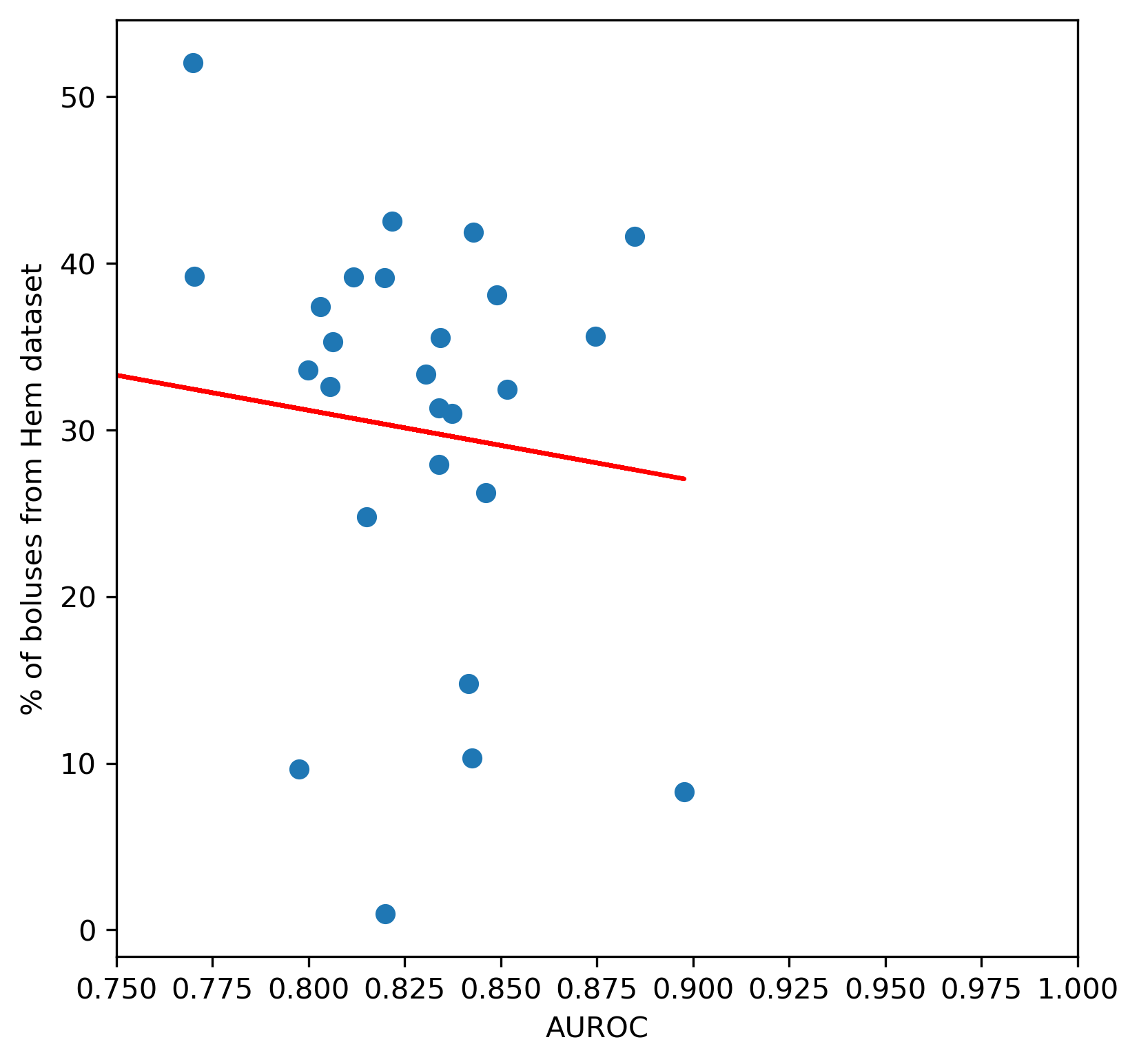
*

**Supplementary Figure 7: Correlation between the proportion of boluses from the hemorrhage injury model in each holdout dataset and model performance.** The plot shows the relationship between the percentage of boluses from the hemorrhagic shock injury model (Hem) in each of the 29 holdout datasets and the corresponding model’s area under receiver operating characteristic curve (AUROC). Data are from models trained using the 12-features consistently retained across feature selection methods.


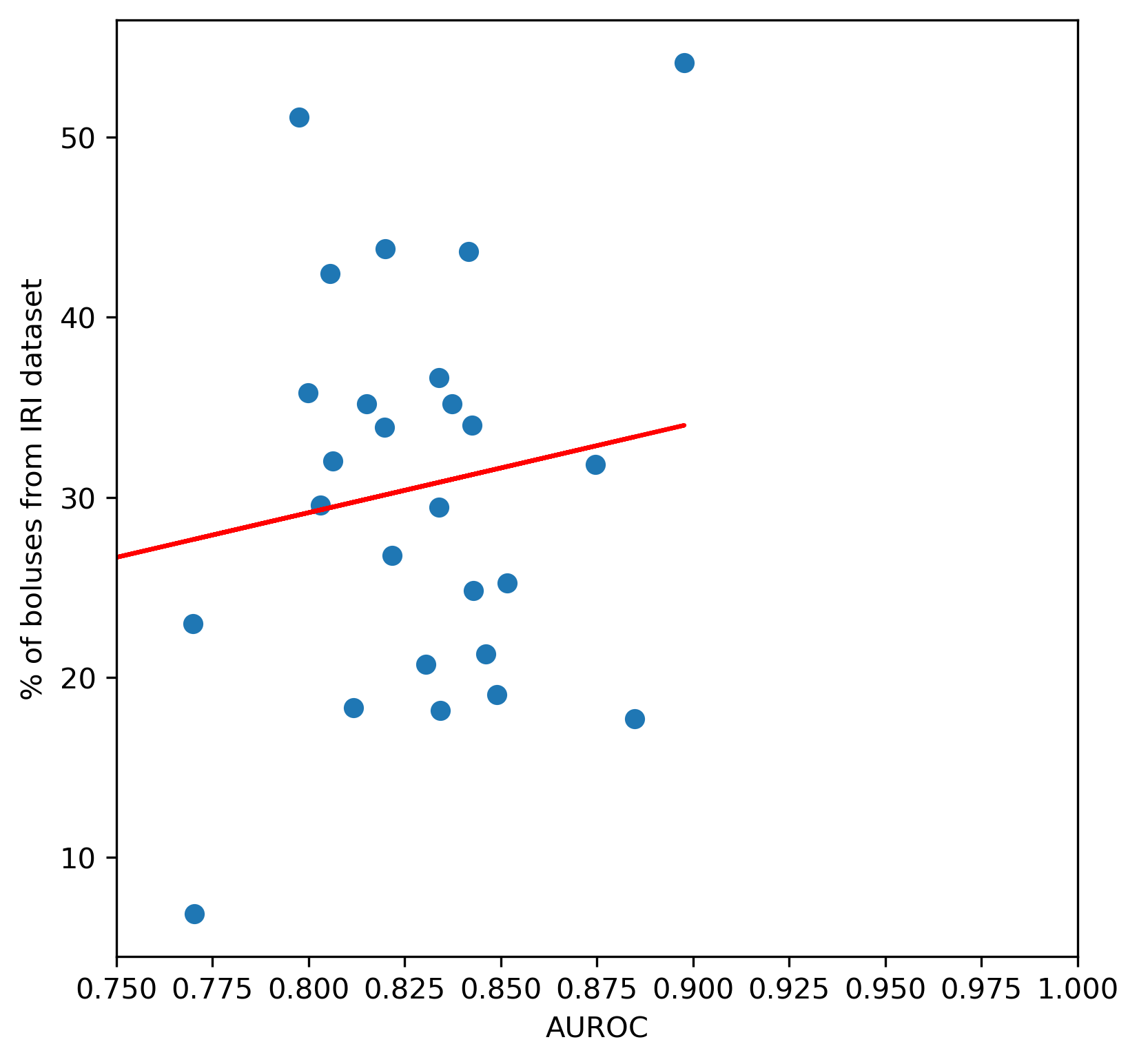


**Supplementary Figure 8: Correlation between the proportion of boluses from the ischemia-reperfusion injury model in each holdout dataset and model performance.** The plot shows the relationship between the percentage of boluses from the ischemia-reperfusion injury model (IRI) in each of the 29 holdout datasets and the corresponding model’s area under receiver operating characteristic curve (AUROC). Data are from models trained using the 12-features consistently retained across feature selection methods.


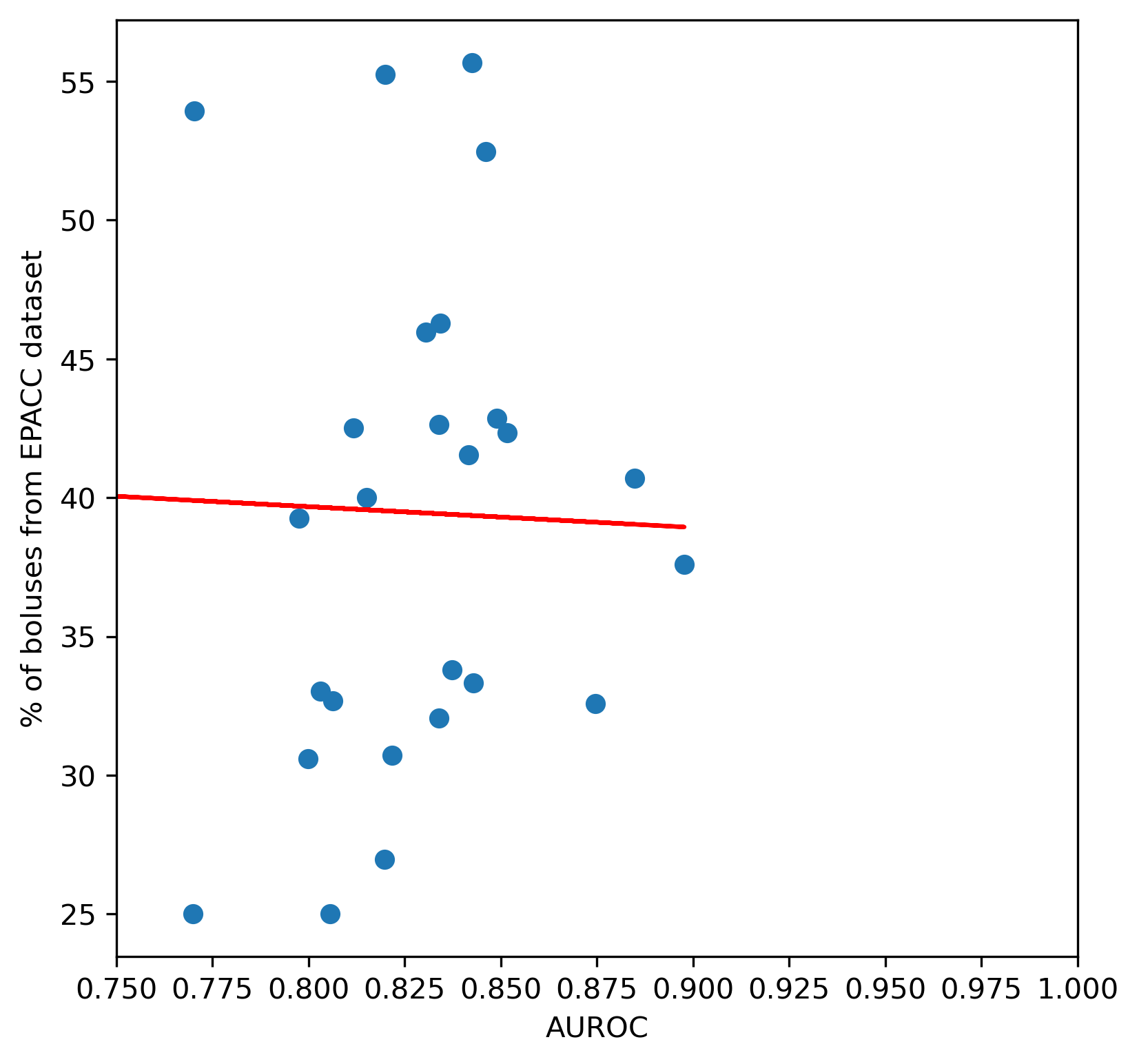


**Supplementary Figure 9: Correlation between the proportion of boluses from the EPACC injury model in each holdout dataset and model performance.** The plot shows the relationship between the percentage of boluses from the EPACC (ischemia-reperfusion injury followed by intermittent occlusion of the supraceliac aorta) injury model in each of the 29 holdout datasets and the corresponding model’s area under receiver operating characteristic curve (AUROC). Data are from models trained using the 12-features consistently retained across feature selection methods.


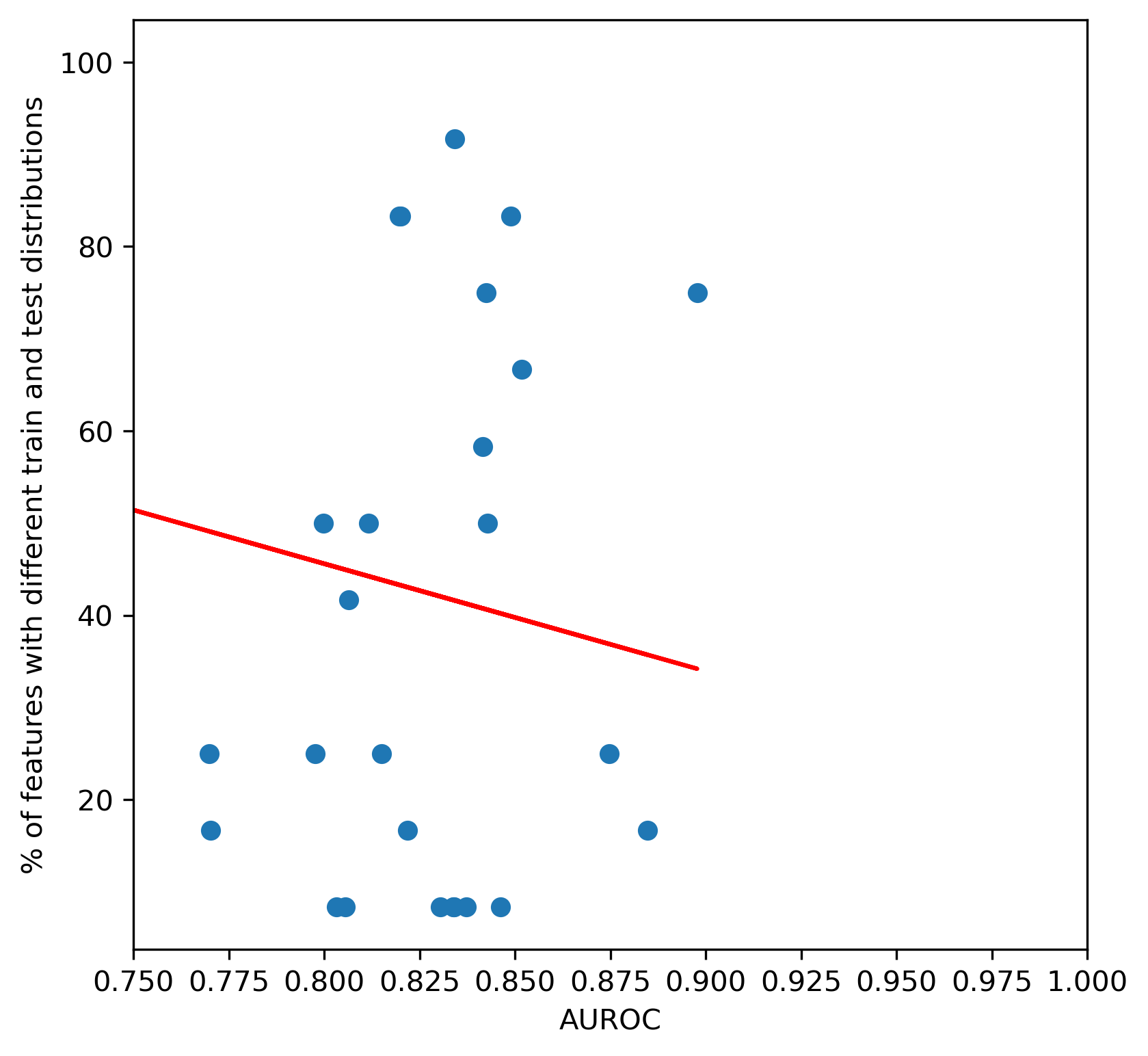


**Supplementary Figure 10: Correlation between the proportion of input features with different distributions between the training and holdout test datasets, and model performance in the holdout datasets.** The plot shows the relationship between the percentage of input features with non-overlapping distributions in each of the 29 train/test splits and the corresponding model’s area under receiver operating characteristic curve (AUROC) in the holdout test datasets. Data are from models trained using the 12-features consistently retained across feature selection methods.
